# Molecular Mechanisms and Clinical Application of Multipotent Stem Cells for Spinal Cord Injury

**DOI:** 10.3390/cells12010120

**Published:** 2022-12-28

**Authors:** Michał Szymoniuk, Jakub Litak, Leon Sakwa, Aleksandra Dryla, Wojciech Zezuliński, Wojciech Czyżewski, Piotr Kamieniak, Tomasz Blicharski

**Affiliations:** 1 Student Scientific Association at the Department of Neurosurgery and Pediatric Neurosurgery, Medical University of Lublin, Jaczewskiego 8, 20-954 Lublin, Poland; 2 Department of Neurosurgery and Pediatric Neurosurgery, Medical University of Lublin, Jaczewskiego 8, 20-954 Lublin, Poland; 3 Department of Clinical Immunology, Medical University of Lublin, Chodźki 4A, 20-093 Lublin, Poland; 4 Student Scientific Society, Kazimierz Pulaski University of Technologies and Humanities in Radom, Chrobrego 27, 26-600 Radom, Poland; 5 Department of Didactics and Medical Simulation, Medical University of Lublin, Chodźki 4, 20-093 Lublin, Poland; 6 Department of Rehabilitation and Orthopaedics, Medical University in Lublin, Jaczewskiego 8, 20-954 Lublin, Poland

**Keywords:** spinal cord injuries, stem cell transplantation, multipotent stem cells, mesenchymal stem cells, neural stem cells, hematopoietic stem cells, regenerative medicine

## Abstract

Spinal Cord Injury (SCI) is a common neurological disorder with devastating psychical and psychosocial sequelae. The majority of patients after SCI suffer from permanent disability caused by motor dysfunction, impaired sensation, neuropathic pain, spasticity as well as urinary complications, and a small number of patients experience a complete recovery. Current standard treatment modalities of the SCI aim to prevent secondary injury and provide limited recovery of lost neurological functions. Stem Cell Therapy (SCT) represents an emerging treatment approach using the differentiation, paracrine, and self-renewal capabilities of stem cells to regenerate the injured spinal cord. To date, multipotent stem cells including mesenchymal stem cells (MSCs), neural stem cells (NSCs), and hematopoietic stem cells (HSCs) represent the most investigated types of stem cells for the treatment of SCI in preclinical and clinical studies. The microenvironment of SCI has a significant impact on the survival, proliferation, and differentiation of transplanted stem cells. Therefore, a deep understanding of the pathophysiology of SCI and molecular mechanisms through which stem cells act may help improve the treatment efficacy of SCT and find new therapeutic approaches such as stem-cell-derived exosomes, gene-modified stem cells, scaffolds, and nanomaterials. In this literature review, the pathogenesis of SCI and molecular mechanisms of action of multipotent stem cells including MSCs, NSCs, and HSCs are comprehensively described. Moreover, the clinical efficacy of multipotent stem cells in SCI treatment, an optimal protocol of stem cell administration, and recent therapeutic approaches based on or combined with SCT are also discussed.

## 1. Introduction

**Figure 1:**
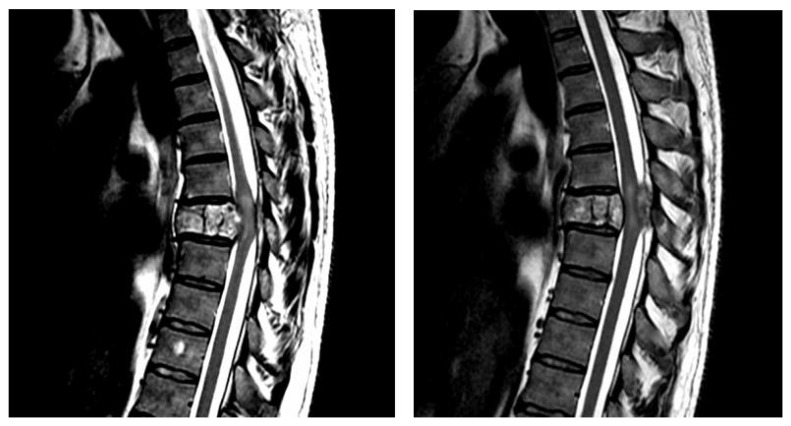
Spinal Cord Injury visualized on MRI-T2 sequence.

Spinal Cord Injury (SCI) is a common neurological disorder with a worldwide incidence ranging from 52 to 56 cases per 1,000,000 people per year and estimated hospitalization costs ranging from $1.6 billion to $1.7 billion per year [1]. This severe neurological condition has devastating physical and psychosocial sequelae. The majority of patients after SCI suffer from permanent disability caused by motor dysfunction, impaired sensation, neuropathic pain, spasticity as well as urinary complications, and a small number of patients experience a complete recovery [2]. Moreover, people with SCI demonstrate from a two to five times higher mortality rate compared with the normal population, which is caused by more frequent kidney failure, respiratory tract infections, and suicides in this population [3]. The severity of motor function impairment mostly affects the prognosis after SCI—motor incomplete injuries demonstrate better treatment outcomes compared with motor complete injuries [4]. The SCI can result from a traumatic as well as non-traumatic etiology. The most common causes of traumatic SCI in developing countries include motor vehicle crashes (43%), falls (34%), gunshot injuries (10%), violence (5%), and sports (2%) [5]. A non-traumatic SCI, a scarcer condition than traumatic SCI, is most frequently caused by degenerative disease, congenital anomalies (e.g. spina bifida, tethered cord), and tumors including primary neoplasms and cancer metastasis [6,7,8,9]. The CT imaging represents the initial diagnostic modality for spinal trauma, whereas the MRI constitutes the gold standard for SCI diagnosis and delivers information about the presence of a spinal cord compression, herniated disc, ligamentous instability, and intramedullary hemorrhage or edema (Figure 1) [10].

The standard treatment of SCI includes hemodynamic support, appropriate hydration, surgical decompression, and subsequent rehabilitation [3]. According to current AO Spine guidelines, surgical decompression and if necessary stabilization should be performed early when possible [11]. It was indicated previously that in patients without contraindications, a 24-h infusion of high-dose methylprednisolone should be administered intravenously within 8 hours after SCI [12]. However, routine methylprednisolone infusion during the acute phase of SCI is not universally accepted and is not recommended [13]. These therapeutic modalities only aim to prevent secondary injury and provide limited recovery of lost neurological functions [14]. Therefore, a plethora of alternative treatment approaches for SCI was presented by many studies in recent years. Numerous studies demonstrated a promising potential of treatment methods modifying the microenvironment of SCI such as betulinic acid, cannabinoids, riluzole, elazanumab, soluble TNF-α receptor 1, and intravenous immunoglobulins [3]. Moreover, recent research focuses on novel therapeutic approaches for spinal cord regeneration such as stem cells, stem cell-derived exosomes, growth factors, nanocarriers, hydrogels, and biomaterial scaffolds [15]. Nevertheless, safe and successful therapy providing complete functional recovery for SCI has still not been established. 

Stem Cell Therapy (SCT) brings new hope for achieving potential neurological improvement of disabled patients after SCI. It represents an emerging treatment modality using the differentiation, paracrine, and self-renewal capabilities of stem cells to regenerate or replace damaged cells and tissues [16]. Numerous reports showed promising outcomes of SCT in the treatment of many conditions including digestive system diseases, liver diseases, dermal wounds, cardiovascular diseases, arthritis, and cancer [16,17,18,19,20,21]. The SCT has been also popularized as a potential treatment for many neurological conditions such as neurodegenerative disorders, multiple sclerosis, stroke, traumatic brain injury, and SCI [22,23,24,25,26]. Regarding the use of SCT for SCI treatment, multipotent stem cells including mesenchymal stem cells (MSCs), neural stem cells (NSCs), and hematopoietic stem cells (HSCs) represent the most investigated types of stem cells for the treatment of SCI in preclinical and clinical studies. The majority of clinical trials investigating SCT for SCI treatment utilized MSCs [27,28,29]. Other stem cells evaluated to date by clinical trials for this purpose include NSCs and HSCs [30,31,32]. Moreover, some clinical research utilized non-stem cell-based therapy and investigated Schwann Cells (SCs), Oligodendrocyte Progenitor Cells (OPCs), and Olfactory Ensheating Cells (OECs) transplantation for SCI treatment with satisfactory results [33,34,35,36]. 

The microenvironment of SCI has a significant impact on the survival, proliferation, and differentiation of transplanted stem cells [37]. Therefore, a deep understanding of the pathophysiology of SCI and molecular mechanisms through which stem cells act may help improve the treatment efficacy of SCT and find new therapeutic approaches based on SCT for SCI treatment. Thus, our literature review aimed to describe the pathogenesis of SCI and molecular mechanisms of action of multipotent stem cells including MSCs, NSCs, and HSCs. The clinical efficacy of multipotent stem cells in SCI treatment, an optimal protocol of stem cell administration, and recent therapeutic approaches based on or combined with SCT are also discussed.

## 2. Pathophysiology of Spinal Cord Injury

The pathophysiology of spinal cord injury is a complex cellular and multimolecular process which can be divided into two major phases: primary and secondary.

The primary stage is a direct consequence of physical and mechanical damage to the spinal cord involving its compression, contusion, shear force, and laceration of the neurons and myelin sheath. The duration and nature of this stage are huge determinants of future recovery [38]. Directly after the initial injury, a cascade of both positive and negative changes starts, including ischemia, disrupted blood flow, proapoptotic signaling, peripheral inflammatory cell infiltration, hyperintensity of glutamate, and regulated cell death, which provokes the extending of primary damage [39,40].

**Figure 2:**
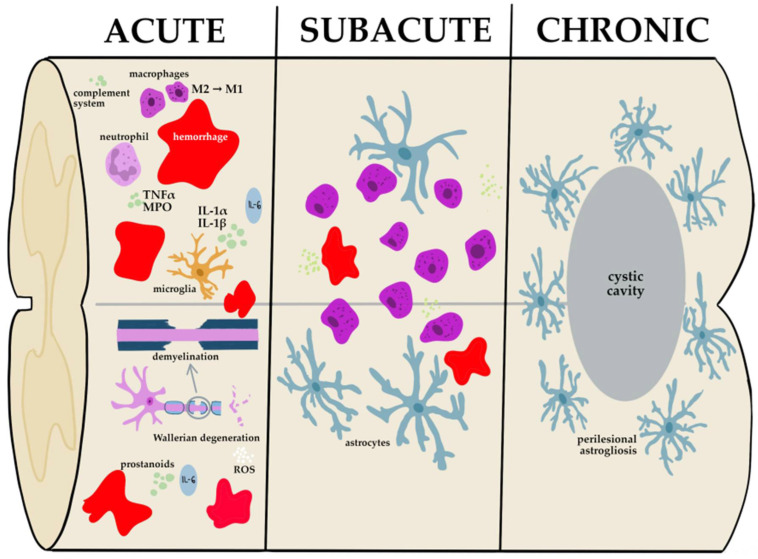
Graphical presentation of the course of SCI secondary stage at the cellular level. Phenomena present in acute SCI (2–48 h): ischemia, mitochondrial failure, ionic imbalance, ROS production, inflammatory processes, Wallerian degeneration, demyelination, glutamate toxicity, debris phagocytosis by macrophages; subacute SCI (2 days–2 weeks): glial scar formation by astrocytes, further debris phagocitosis; chronic SCI (>2 weeks): glial scar maturation, cyst formation, axonal sprouting.

The secondary stage can be divided into three subgroups: acute, subacute (intermediate), and chronic stage in terms of time from injury (Figure 2) [41]. The first stage of secondary injury lasts from 2 to 48 h. Ruptured vessels and the destroyed blood-spinal-cord barrier result in cytotoxic and vasogenic edema and hemorrhage into the parenchyma of the spinal cord, especially into the white matter which can provoke cytotoxic and vasogenic edema [42,43]. The red blood cells present in extravasated blood undergo destruction after time which leads to a toxic accumulation of iron ions in near tissue. This leads to ferroptosis of local cells which is a non-apoptotic, iron-regulated kind of cell death when iron overload activates the reactive oxygen species generation, dysregulation of the glutathione/glutathione peroxidase 4 (GSH/GPX4) metabolism, and accumulation of lipid peroxides, which cause lipid membrane deterioration [40]. 

Swelling of the axons may co-occur with Wallerian degeneration, but its etiology remains uncertain [44]. Subsequently, the disintegrated blood-spinal-cord barrier facilitates the entry of immune cells, such as macrophages, T cells, microglia, and neutrophils, which triggers the release of inflammatory cytokines such as tumor necrosis factor-α (TNF-α), interleukins (IL-1α, IL-1β, and IL-6), nitric oxide (NO^•^), reactive oxygen species (ROS), elastase, and matrix metalloproteinase-9 (MMP-9) [38,45].

The interrupted blood-spinal-cord barrier facilitates the excessive influx of water into the extracellular compartment resulting in edema and ion imbalance. Ionic dysregulation is characterized primarily by a Na^+^ and Ca^2+^ intracellular concentration with a simultaneous elevated extracellular concentration of K^+^ and Mg^+^ [39]. Intracellular hypercalciuria activates calcium-dependent proteases and causes mitochondrial dysfunction ultimately leading to apoptotic cell death [38]. 

Membrane depolarization leads to the release of glutamate into the extracellular milieu which is relevant to neurotransmitter deregulation. The glutamate binds to an extrasynaptic receptor NMDAR which causes neuronal excitotoxicity by the receptor-mediated influx of calcium into the cell [46]. All formation processes may contribute to forming free radicals such as NO^•^, OH^−^, and H_2_O_2_ which can bind with the cell’s molecules and oxidize them.

During chronic and sub-acute phases, apoptosis and necrosis of neurons occur as a consequence of prior cellular and intercellular changes. The glial scar formation is a multifactorial phenomenon that involves oligodendrocyte precursor cells, pericytes, microglia fibroblasts, chondroitin sulfate proteoglycans, and particularly activated astrocytes [45]. Activated astrocytes lead to astrogliosis which is a defense response of the central nervous system to minimize and repair primary damage, but it eventually generates harmful effects due to producing high levels of inhibitory molecules to suppress neuronal elongation and forming potent barriers to axon regeneration [47,48]. 

## 3. Stem Cell Types for Stem Cell Therapy

### 3.1. Stem Cells’ Classification

To understand the characteristics of each type of stem cell used for SCT better, we should know their origin and differentiation potential into various cell types. Regarding the origin of stem cells, they can be divided into two major categories—adult stem cells and embryonic stem cells [49,50]. Based on the range of their differentiation potential, stem cells can be categorized into five classes: totipotent, pluripotent, multipotent, oligopotent, and unipotent [51]. Totipotent activity implies the capability of differentiation into any type of an organism’s cells including placental cells and three germ layers, and is demonstrated only by embryonic stem cells (ESCs) derived from morula (1–3 days after fertilization) [49,50]. On the other hand, ESCs obtained from a blastocyst (4–14 days after fertilization) demonstrate pluripotent activity which indicates the capability of the generation of all types of cells in the body excluding placental cells [49,50]. Pluripotent cells can be also sourced from extra fetal tissues such as the umbilical cord, amniotic fluid, amnion, and chorion [49]. Furthermore, pluripotent stem cells can be generated from adult somatic cells using so-called OSKM transcription factors which include OCT-4, SOX2, KLF4, and c-MYC [52]. Created through that genetic reprogramming of stem cells namely induced pluripotent stem cells (iPSC) demonstrate embryonic-like molecular and biological features [16]. Another type of differentiation potential, multipotency, implies the ability to transform into a limited number of specific cell types [49,51,53] Multipotent stem cells are undifferentiated, self-renewing cells including several stem cell types in an adult organism such as those present in bone marrow mesenchymal stem cells (MSCs) and hematopoietic stem cells (HSCs), or neural stem cells (NSCs) [54]. The MSCs can generate adipocytes, bone, and chondrocytes, whereas HSCs can differentiate into all cell types of the hematopoietic system [53]. However, it was demonstrated that adult stem cells can also form cells from other cell lineages depending on molecular signals from the microenvironment where they were transplanted [55]. That phenomenon called stem cell plasticity significantly expanded its potential use for the treatment of many diseases, including SCI. Furthermore, oligopotent stem cells have a narrower differentiation spectrum and can transform only into several cell types of a specific tissue (e.g., myeloid cells which can differentiate into leukocytes but not erythrocytes) [51]. Finally, unipotent stem cells can form only one cell type, but compared with non-stem cells they have a self-renewal capability [51,53]. 

### 3.2. Pluripotent Stem Cells

The pluripotent stem cells including ESCs and iPSCs, as unlimited self-renewable cells, represent promising types of stem cells for treatment replacing damaged tissues. 

Under specific conditions, the ESCs can generate any cell lines, e.g., neurons or oligodendrocytes [56]. Thus, several studies utilize ESCs-derived stem cells or ESCs-derived extracellular vesicles [57,58,59]. Currently, an ongoing clinical trial evaluates safety and efficacy of the transplantation of neural precursor cells (NPCs) derived from human ESCs for AIS-A, sub-acute SCI patients (NCT04812431). However, some major limitations hamper the introduction of ESCs into clinical trials due to obtaining them from non-autologous blastocysts such as the risk of immune rejection and ethical concerns regarding the use of human embryos [16]. Thus, recent research tries to develop effective technology generating ESCs such as nuclear transfer technology, which may avoid these problems [16,60]. Moreover, the high differentiation potential of ESCs is associated with the risk of tumorigenicity, especially the possibility to form teratomas [61].

Artificially generated iPSCs avoid ethical problems associated with ESCs harvested from human embryos and maintain the beneficial capabilities of ESCs [62]. Moreover, iPSCs similarly to ESCs may be utilized as a source to generate multipotent stem cells for transplantation, e.g., neural stem cells [63]. However, the use of iPSCs is also faced with major challenges such as immune rejections, the instability of iPSCs’ genome, and potential tumorigenicity [64,65,66]. To date, there are no published clinical trials regarding the use of pluripotent stem cells for SCI treatment.

### 3.3. Multipotent Stem Cells

Mesenchymal Stem Cells or Mesenchymal Stromal Cells (MSCs) are multipotent progenitor cells, which exhibit the greatest potential for treating spinal cord injury among all stem cell types [67]. MSCs are characterized by easy extraction, and rapid proliferation and can be obtained from the patients themselves [68,69,70]. MSCs for clinical applications can be generated from autologous sources, such as bone marrow and adipose tissue [71]. Alternatively, there are allogeneic sources of MSCs, which include umbilical cord blood, placenta, and amniotic fluid [14,72]. MSCs are characterized by low immunogenicity, and bone marrow MSCs (BMSCs) cause the least intensified immunologic response among MSCs from mentioned sources [73,74]. In comparison to BMSCs, adipose-derived stem cells (ADMSCs) exhibit three times higher activity and are easily available for obtainment [75]. Both ADMSCs and BMSCs can be generated without ethical issues, but it requires liposuction or bone marrow aspirate followed by cultivation, which makes them time-consuming and expensive sources [14,72,76]. On the other hand, Umbilical cord or Wharton’s Jelly MSCs (UCMSCs) are easier to obtain, but require conducting complex procedures namely lyophilization to avoid immunological responses and are controversial from the ethical point of view [74]. Besides that, UCMSCs are characterized by fast proliferation, low immunogenicity, and faster in vitro expansion than the other MSCs [77,78]. The MSCs have been investigated for SCI treatment in the greatest number of clinical trials among stem cell types so far.

Recently, the NSCs were introduced into clinical trials and showed promising results for application in the treatment of the injured spinal cord. As of today, Neural Stem Cells can be obtained from three distinctive sources courtesy of recent technological advances. NSCs can be derived either from primary tissues, as means of differentiating them from pluripotent stem cells or via trans differentiation from mature somatic cells. As for isolating NSCs from primary tissue, it was proven that NSCs can grow in single-cell suspensions, stimulated by the epidermal growth factor (EGF) and basic fibroblast growth factor (bFGF). These cells derived from, e.g., periventricular regions by means of cell sorting based on expressed NSCs’ markers, as is the case for mammals, although no protocol yet has been obtained for this type of procedure in humans, so it can be considered as an ethically ambiguous endeavor. An alternative from primary tissue extraction is the differentiation of pluripotent stem cells, such as patient specific in iPSCs derived from reprogrammed skin fibroblasts [79]. Neural Stem Cells can be potentially derived from fetal CNS (central nervous system) tissue, such is the case with HuCNS-SC, Stemcells, Inc, Newark, CA. HuCNS-SC was proven safe for intraspinal transplantation at high doses by studies classified at class IV evidence [79]. As for implantation of the autologous human Schwann cells with SCI, there was no evidence of additional spinal cord damage, mass lesion, or syrinx formation [80]. One other aforementioned method is the trans differentiation of somatic cells. This method essentially transforms mature somatic cells of one type into another utilizing exogenous transcription factors. Such was the case with zinc-finger transcription factor, Zfp521. Research has given us a way for direct conversion of human fibroblasts into long-term self-renewable and multipotent NSCs [81]. Another way of obtaining NSCs from fibroblasts without the need for genetic manipulation is cellular reprogramming using pharmacological methods. M9, a chemical cocktail developed by Zhang et al., was shown to reprogram mouse fibroblasts into induced neural stem cell-like cells (ciNSLCs) [82]. These cells show great promise, as they resemble primary NCS in terms of self-renewal and differentiation capabilities, although more research has to be conducted in order to understand the process fully and implement these methods in human research models.

The HSCs exhibited safety for clinical use and were investigated with satisfactory outcomes as a treatment for many diseases such as hematopoietic diseases, multiple sclerosis, Crohn’s disease, and diabetes danielson, mohammadi oliveira [83,84,85,86]. The HSCs can be harvested from the placenta, cord blood, and adult bone marrow at acceptable concentration levels [61]. However, umbilical cord blood contains a significantly higher amount of HSCs than bone marrow, and umbilical cord-derived HSCs are characterized by lower immunogenicity than bone-marrow-derived ones [87]. Indeed, immune rejection constitutes the most challenging concern associated with the use of HSCs [88]. Nevertheless, treatment with HSCs is devoid of tumorigenic complications [89]. Moreover, the Food Drug Administration (FDA) approved the HSCs for stem cell therapy in patients with conditions that affect the hematopoietic system [90,91]. To date, HSCs in this setting constitute only one type of stem cell approved by the FDA. Regarding the use of HSCs for SCI therapy, the results of several clinical trials have been published to date.

In the following sections, special attention is paid to multipotent stem cells including MSCs, NSCs, and HSCs as regards their molecular mechanisms and clinical aspects of their use for SCI treatment. Table 1 summarizes the types of stem cells used for SCT regarding the sourcing, differentiation potential, advantages, and limitations (Table 1).

## 4. Molecular Mechanisms of Multipotent Stem Cells at SCI Microenvironment

### 4.1. Mesenchymal Stem Cells

MSCs reach the lesion site through the chemotactic mechanism known as a homing effect. This phenomenon is relevant for therapeutic efficacy not only in the case of the intrathecal and intravenous routes of administration but also in intralesional injection [71]. According to recent studies, many factors are involved in these mechanisms. The SDF-1/CXCR4 (Stromal-cell derived factor-1/CXC chemokine receptor 4) signaling pathway has a significant regulatory role in the homing effect, and its upregulation may improve the migration of MSCs to the injury site [93,94,95]. Inflammation, hypoxia, and ischemia, conditions characterizing the SCI microenvironment, especially in the acute phase, elevate the expression of SDF-1 [96]. Binding SDF-1 (also known as CXCL12) to CXCR4, the surface receptor of MSCs, leads to activation of signaling molecules such as ERK, PI3K, and Akt, attracting MSCs to the lesion site [97]. Other important factors, which stimulate migratory behavior of MSCs, include substance P, aquaporin 1, calcitonin gene-related peptide (CGRP), and a variety of growth factors such as the granulocyte colony-stimulating factor (G-CSF), vascular endothelial growth factor (VEGF), basic fibroblast growth factor (bFGF), leukemia inhibitory factor (LIF), and hepatocyte growth factor (HGF) [71,72,94,98,99,100,101,102]. Interestingly, substance P impairs the migration of MSCs in response to TGF-β [103]. However, the precise mechanisms determining the homing capacity of MSCs remain unclear. 

The differentiation potential of MSCs demonstrated by in vitro studies brought great hope for their use in SCI treatment as a cellular replacement for damaged neural cells. In these experiments, MSCs differentiated into neural lineages showed some electrophysiological properties and expressed proteins characteristic of nerve cells [96,104]. However, despite the neuron-like phenotype of differentiated MSCs, these cells were unable to activate action potentials [96]. Moreover, in vivo studies demonstrated a limited differentiation ability of MSCs. Transplanted MSCs did not show specific electrophysiological activity, and their survival number was too small to provide regeneration of damaged structures [71,105,106] Therefore, the differentiation capability of MSCs probably plays a secondary role in functional recovery in patients with SCI. Indeed, data from many studies indicate that benefits provided by SCI therapy rather result from the paracrine and immunomodulatory activity of MSCs than their trans differentiation into the neural cells [107,108].

The paracrine effect of MSCs relies on secreting multiple cytokines, growth factors, and other bioactive molecules, which are contained in MSCs’ exosomes and microvesicles [109]. These substances stimulate neuronal and tissue regeneration, reduce glial scarring, enhance angiogenesis, regulate inflammatory processes, and modulate immune responses [109,110]. The secretome of MSCs include the nerve growth factor (NGF), brain-derived neurotrophic factor (BDNF), glial cell-derived neurotrophic factor (GDNF), ciliary neurotrophic factor (CNTF), vascular endothelial growth factor (VEGF), insulin-like growth factor-1 (IGF-1), basic fibroblast growth factor (bFGF), hepatocyte growth factor (HGF), platelet-derived growth factor (PDGF), pigment epithelium-derived factor (PEDF), tissue inhibitor of metalloproteinase-1 (TIMP-1), glia-derived nexin (GDN), interleukin-6 (IL-6), interleukin-8 (IL-8), neurotrophin-1 factor (NT-1), neurotrophin-3 factor (NT-3), galectin-1 (Gal-1), and cystatin C [72,96,111]. Several studies demonstrated that MSCs can exert neuroprotective activities including counteracting nerve degradation and supporting neurogenesis, oligodendrogenesis, remyelination, and axonal growth [72]. The substances secreted by MSCs responsible for those capabilities include BDNF, GDNF, HGF, TIMP-1, NT-1, NT-3, bFGF, and CNTF [71,72]. BDNF, a neurotrophin, is one of the key molecules engaged in neuronal development in CNS [112]. In a spinal cord injury environment, BDNF increases the volume of nerve tissue and decreases the area of the cystic cavity [113]. BDNF achieves a neuroprotective effect probably through activation of the Akt pathways and through its high-affinity tropomyosin-related kinase type B (TrkB.FL) receptor [114]. GDNF has a potentially significant role in the reduction in secondary injury and motor recovery [115,116]. GDNF also demonstrated antioxidative properties by stimulating the enzymes responsible for the neutralization of reactive oxygen species [96]. Moreover, GDNF enhances the survival of grafted MSCs and promotes axonal growth [116,117]. Another growth factor, HGF, through the c-Met receptors, increases axonal growth, promotes angiogenesis, decreases glial scar formation, and inhibits demyelination, blood-brain barrier impairment, and apoptosis [71,118]. Noteworthy, c-Met receptors are overexpressed during the acute phase of spinal cord injury [118]. Furthermore, TIMP-1 secreted by MSCs has demonstrated the capability of oligodendrogenesis stimulation [119]. 

The glial scar constitutes a barrier that inhibits axonal growth and regeneration after SCI [120]. Transplantation of MSCs in a rat SCI model demonstrated reduced glial scar formation and increased axonal regeneration [121]. In this phenomenon, the paracrine activity of MSCs also plays a significant role. Indeed, transplantation of human UCMSCs overexpressing bFGF to a mouse SCI model improved neural regeneration and glial scarring through the activation of the PI3K-Akt-GSK-3β pathway [122]. Moreover, reduction in the levels of TGF-β through HGF secretion by MSCs also suppressed glial scar formation [123]. Furthermore, MSCs can inhibit the TGF-β/Smads signaling pathway in astrocytes, which is also involved in glial scar formation [124]. The modulation of astrogliosis via the matrix metalloproteinase-2/signal transducer and activator of transcription 3 (MMP-2/STAT3) signaling pathway is the other important mechanism responsible for suppressing glial scarring by MSCs [71,125]. Inhibiting glial scar formation is beneficial for neural repair in subacute and chronic SCI. However, in the acute phase of SCI, the suppression of glial scarring may increase the spread of various inflammatory cells and toxic molecules from the lesion site [126]. A study on the SCI rat model showed that MSCs decreased glial scarring in a chronic stage of SCI and increased the formation of glial scar in the early stage, but this observation should be confirmed in further studies [127].

Angiogenesis induction at the lesion site is an especially important capability in supporting spinal cord injury healing [128,129]. This phenomenon is carried out through secretion by MSCs with the molecules such as VEGF, PDGF, bFGF, HGF, IGF-1, GDNF, BDNF, TIMP, IL-6, and IL-8, which are responsible for creating new vasculature from pre-existing vessels [72,96,111]. Angiogenesis stimulation facilitates axonal regeneration, improves ischemia, and hypoxia, and prevents accumulation of inflammatory molecules at the injury site [96,128]. 

The immune reactions after SCI are thought to be one of the most significant secondary injury factors [130]. At the lesion site, transplanted MSCs exert immunoregulative function through suppression of the inflammatory response, inhibition of T cells, and reprogramming of the microglia phenotype [71]. Studies showed that MSCs reduce levels of inflammatory cytokines including TNFα, IL-1β, IL-2, IL-4, IL-6, and IL-12 at the injury site [131]. In these phenomena, paracrine activity of MSCs also has substantial relevance and includes cytokines and trophic factors such as CNTF, TNF-beta1, neurotrophin 3 factor (NT-3), IL-18 binding protein, and interleukins (IL-13, IL-10, IL-12p70, IL-17E, IL-27) secreted by MSCs [72]. Moreover, MSCs transplanted into the lesion site maintain MHC-I, Sca1, and CD29 expression levels on their surface and additionally boost their expression of MHC-II and CD45, which means that MSCs adopt the immune cell-like phenotype in response to the SCI microenvironment [132]. Probably, interferon-gamma (IFNγ) present in a SCI environment is mainly responsible for the induction of MHC-II expression by MSCs [38,132]. Moreover, exposure to IFNγ and TNF-α triggers anti-inflammatory properties in MSCs through induction of indoleamine 2,3-dioxygenase (IDO1), IL-4, IL-10, CD274, and PD-L1 expression [96]. MSCs may also inhibit the proliferation and activation of T cells through the promotion of p27Kip1 expression and decreasing of the cyclin D2 expression, which results in the arrest of the cell cycle at the G1 phase [133]. This process is mediated by many molecules including TGF-β1, PGE2, HGF, IDO1, and NO [134]. MSCs may also inhibit Th1 and Th17, while at the same time promoting the formation of Treg and Th2 cells [135]. Furthermore, MSCs inhibit neurotoxic A1 astrocytes probably through inhibiting the nuclear translocation of phosphorylated nuclear factor kappa B (NfκB) pathway p65 subunit [136]. The inflammatory reaction is inhibited by MSCs also by increasing the M2 polarization of macrophages and decreasing the M1 macrophage polarization [137,138]. M1 mainly produces pro-inflammatory cytokines including TNF-α, IFN-γ, IL-1β, IL-6, IL-12, and IL-23, whereas M2 releases immunosuppressive molecules such as IL-4, IL-10, IL-13, and TGF-β promoting tissue repair [139,140,141]. IL-10 secreted by MSCs is considered one of the key factors responsible for the transformation of the macrophage phenotype through activation of the JAK/STAT3 signaling in macrophages [123].

### 4.2. Neural Stem Cells

NSCs are self-renewing, multipotent cells that can give rise to neurons, astrocytes, and oligodendrocytes. They can be observed in states of dormancy and mitotic activation, depending on the parameters of their environment. Neural Stem Cells tend to express low levels of extracellular matrix receptors in their dormant state, but, when they become mitotically active, receptors such as integrin- α6β1, syndecan-1, and Lutheran have a much higher expression [142]. As for outside components, a family of proteins known as BMP (bone morphogenic proteins) plays a role in the proliferation and differentiation of NCS. LRP2, a receptor for BMP4 for example, is theorized to be crucial in their proliferation, as research shows that in mice without this receptor, neural progenitors cease to proliferate. When BMP secretion inhibitors’ overexpression was tested, specifically the Noggin, NSC enhanced their proliferation of progenitors and shifted SVZ lineage progression from mature astrocytes to transit amplifying cells and oligodendrocyte precursors. Noggin also promoted the differentiation of both oligodendrocytes and neurons, which was inhibited by BMP4 [143]. Other molecules that have been shown to upregulate NSCs’ proliferation in the subependymal zones such as Ansomin-1 binding to FGFR1, as well as induce their migration [144]. A crucial part of NSCs’ research is finding novel molecules that orient them in their environment and allow them to connect into more complex chains, such is the case with Epherin-A and B signaling pathways. Research finds that especially EphA4 suppression causes the population of neuroblasts and astrocytes to become loosely aligned and chaotic, often migrating into neighboring structures [145]. NSCs and progenitor cells descended from them express Wnt receptor FZD1 playing a similar role, as the knockout of FZD1 was prioved to cause astroglial differentiation with increased migration of adult-born neurons but also a shutdown of new neuron differentiation [146]. 

Neurotransmitters abundant in the regions of the NSC residency also play a major role in shaping stem cells. The best-described example of regulating neurogenesis, particularly in the SEZ region is gamma-aminobutyric acid. GABAergic neurons were proven to control NSC populations by maintaining their status of quiescence in the hippocampus [147]. Neurogenesis stemming from choline acetylase was explored in rodent SVZs where a stroke was experimentally induced; a population of ChAT-positive neurons was found to have participated in the proliferation of NSCs and their homing to zones damaged by the stroke, resulting in better recovery [148]. 

A neurotransmitter that induces NSCs’ activity is norepinephrine via the β3 adrenergic receptors.

Ghrelin administration was proven to induce cellular proliferation of hippocampal NSC via such pathways as ERK1 and 2, as well as PI3K, and Janus kinase 2 [149]. Melatonin was proven to facilitate fetal bovine serum-induced neural differentiation of NSCs without affecting the astroglial differentiation [150]. 

### 4.3. Hematopoietic Stem Cells

HSCs as multipotent stem cells can differentiate into all types of blood cells and lymphoid lineages [151]. Transplanted into the SCI microenvironment, HSCs exert their therapeutic activity through differentiation and releasing numerous cytokines and neurotrophic factors. 

The differentiation capacity of HSCs at the SCI microenvironment includes transforming into astrocytes, neuroprotective glia, and oligodendrocytes [152]. In a recent in vitro study, human umbilical cord blood-derived CD133^+^ HSCs after exposure to the mixture of sonic hedgehog, BDNF, B27, and retinoic acid demonstrated increased expression of Isl-1, AchE, SMI-32, and Nestin, which are markers specific for motor neurons [153]. That suggests the potential of HSCs for differentiation into motor neuron-like cells.

Preclinical studies showed that a plethora of growth factors and cytokines could be released by HSCs including VEGF, thrombopoietin, neurotrophin-3 (NT-3), mitogen-activated protein kinase-1 (MEK-1), angiopoietin-1, IL-11, and colony-stimulating factor I (CSF-I) [89,154,155]. An animal study by Xiong et al. demonstrated that the administered in the chronic phase of SCI HSCs increased expression levels of NT-3 and MEK-1 suggesting that HSCs exert their neuroregenerative properties trough release mainly of these two factors [154]. The signaling pathways that involve MEK-1 and NT-3 play important roles in neuroprotection and are significantly downregulated after SCI, which indicates that HSCs restore proper MEK-1 and NT-3 levels [156,157]. Moreover, inhibition of astrogliosis, enhancement of 5-HT-positive fibers, and oligogenesis promotion after HSCs’ administration were also observed [154]. Suppressing astrogliosis inhibits the formation of a glial scar at the lesion site. As above mentioned, the benefits coming from inhibition or promotion of glial scarring may vary regarding the phase of SCI. Therefore, inhibition of astrogliosis at the chronic stage of SCI unleashes regenerating axons from suppressive effects of inhibitory molecules and fibrotic scarring [154], whereas, during the acute phase of SCI, promotion of astrogliosis may be beneficial due to the protective role of the glial scar against the inflammatory environment of acute SCI [154]. On the other hand, stimulation of oligogliosis regenerates demyelinated axons, and enhancement of 5-HT fibers extends their lateral branches, which enhances neural improvement [154]. 

An exact molecular mechanism of action through which HSCs exert their neuroregenerative properties in the treatment of SCI remains not thoroughly investigated; thus, further studies are needed to unveil other molecular interactions involved in their activity.

## 5. Clinical Studies Regarding Multipotent Stem Cells for SCI Treatment

### 5.1. Mesenchymal Stem Cells

Among stem cells proposed for SCI treatment, MSCs are most investigated in clinical studies and show a high potential for their use in this purpose. The safety of their transplantation was demonstrated in many preclinical and clinical studies [158,159,160,161].

However, the efficacy of MSCs in SCI treatment remains unclear due to the lack of well-designed, randomized, controlled studies on a large group of patients. To date, the majority of clinical research is represented by one or two phases of clinical trials with limited study populations. A non-randomized clinical trial by Oh et al. is the only published phase 3 clinical trial [68]. This study included a small number of patients (16), whereas two of them showed motor improvement. However, these patients have an incomplete injury and underwent a standard rehabilitation program; thus, the possibility of spontaneous improvement is high [162]. Recent clinical studies regarding SCT for SCI are presented in detail in Table 2.

So far, most clinical studies focus on the use of BMSCs for SCI therapy. A recent randomized placebo-controlled trial by Saini et al. evaluated the clinical effectiveness of intramedullary administered BMSCs for 13 patients with acute complete SCI [27]. Only sensory function was improved from a mean ASIA score of 124 to 224 at 6 months in comparison to controls with a static mean of 115. Motor functional improvement has not been achieved in any of the patients. Interestingly, in a network meta-analysis by Liu et al., BMSCs combined with rehabilitation demonstrated significant improvement compared with rehabilitation training in the ASIA impairment scale grade, ASIA motor score, ASIA sensory functional score, and Barthel Index [172]. However, the weighted mean difference (WMD) for the ASIA motor score achieved the lowest value (6.67; 95% CI, 0.83–12.73). A meta-analysis by Chen et al. showed comparable data [173]. Other conducted meta-analyses obtained similar results indicating that only mild sensory or bladder function improvement is observed after MSCs’ transplantation without significant motor function recovery [74,174,175,176]. The safety and efficacy of BMSCs for SCI treatment remain under further investigation by registered conducting clinical trials (NCT01162915, NCT02981576, NCT02570932, NCT04288934, NCT01909154, NCT01325103).

Regarding ADMSCs, there is only one published clinical study so far. This study by Hur et al. demonstrated minimal improvement only in 5 of 14 patients 8 months after intrathecal administration of 9 × 10^7^ ADMSCs per patient [29]. A limited number of patients, administration of ADMSCs a long time after injury, and including patients with incomplete injury might have influenced these results. Currently, recruiting the 1/2 phase clinical trial (NCT02917291) will evaluate the safety and potential efficacy of FAB117-HC (a product containing human HC016 cells generated from expanded allogeneic adipose-derived MSCs and pulsed with H_2_O_2_) for acute SCI. The oxidative environment is regarded as a major limitation for MSCs’ engrafting; thus, the addition of H_2_O_2_ may resolve this problem [177]. Other ongoing clinical trials currently evaluating the efficacy of ADMSCs for SCI include NCT04520373, NCT03308565, NCT05018793, and NCT02981576.

To date, there is also a limited number of studies investigating the use of UCMSCs for spinal cord injury. In the phase 1/2a randomized controlled trial, sensory improvement was observed in patients with complete chronic SCI after intrathecal administration of Wharton jelly-derived MSCs. [14] However, no changes in motor function have been observed, which is consistent with the results of studies previously discussed in this section. Moreover, in a meta-analysis by Liu et al., UCMSCs combined with rehabilitation have not demonstrated significant differences in clinical outcomes compared with rehabilitation alone or UCMSCs alone [172]. Currently conducting clinical trials evaluate multiple administrations of UCMSCs (NCT02481440), the safety and efficacy of UCMSCs (NCT05152290, NCT03003364), and compare them with BMSCs (NCT04288934).

### 5.2. Neural Stem Cells 

When discussing novel NSC therapies, one must take into consideration the safety of injecting stem cells into the spinal canal. Recent studies proved that the safest way of delivery is via perilesional intramedullary injections, under the guidance of ultrasound imaging, to the immediately adjacent spinal segment that presented an abnormal SSEP/MEP signal after exposing the dural opening. Injections of marked depth from 3 to 4 mm, previously calculated by the pre-procedural MRI, were deemed as a successful site for transplanting a total dose of 40 M HuCNS-SC using a free-hand technique [167].

Research published in 2020 had reassuring long-term results of the same procedures. A total of 20 M HuCNS-SC cells were transplanted to 12 participants. A six-year follow-up clinical assessment consisting of neuroimaging and a sensory threshold found short- and long-term safety for NSC therapy [30]. 

A study conducted in 2018 deemed the first in human phase I study of neural stem cell transplantation for CSCI with a small subject pool shows promising results. Four subjects received NSI-566 spinal cord injections of NSCs after assessing their ISNCSCI scores, functional and pain surveys, SCIM scores, EMGs, BMCA, and MRIs. In the following 6, 12, 18, and 27 months, these tests were reconducted yielding results of slight motor function and sensory improvement in three of four cases. Unfortunately, due to the lack of a control group and a small number of subjects, the study is not decisive, yet it paves the way for future research [168]. 

A second study aimed at assessing the safety and feasibility of HuCNS-SC transplants for chronic SCI was concluded and published in 2018. Totals of 11 patients in the research group and 13 in the control group were analyzed against each other. The research group was given the aforementioned HuCNS-SC cellular product, using established free-hand techniques in accordance to the current state of knowledge. The results yielded improvements in UEMS and GRASSP strength for 6 months in GASSP with a decline to the baseline control group in 9 months’ time. The UEMS score showed an improvement of 2.83 points at 9 months. Unfortunately, the research was halted due to funding issues [31].

As of today, there is only a handful of ongoing research projects that try to utilize NSCs in SCI in human subjects. Safety Study of Human Spinal Cord-derived Neural Stem Cell Transplantation for the Treatment of Chronic SCI was implemented on patients who suffered from SCI injury classified as AIS-A in the period between 1 and 2 years from the study’s beginning. The patients were separated into two groups, one with 4 patients with spinal cord injuries diagnosed at the T2-T12 level and another with 4 subjects at the C5-C7 level. Graft survival in the transplant site was determined by MRI (for Group A) and via autopsy, if one was completed. The patients then went through an evaluation of the ability of HSSC transplantation positively to affect the AIS level ISNC SCI motor and sensory index scores, bowel and bladder function, pain, UAB IMR scores, SCIM scores, evoked sensory and motor potentials, and electromyogram (EMG). The outcomes of this specific clinical trial have not been published (NCT01772810).

Safety and Exploratory Efficacy of Transplantation Therapy Using PSA-NCAM(+) NPC in the AIS-A Level of Sub-acute SCI was also aimed to evaluate these parameters using neural precursor cells derived from embryonic stem cells. Test subjects were selected among C4-C7 AIS-A diagnosed patients and administered with PSA-NCAM (+) PC to explore the Dose Limiting Toxicity in triplets, adding two patients to the study if no DLT effect is observed. The cells are administered through intrathecal injections to five areas in each patient. The study is currently recruiting at Ajou University Hospital in Korea and estimated the completion date around September 2028 (NCT04812431). 

Umbilical Cord Blood Cell (MC001) Transplant Into Injured Spinal Cord Followed by the Locomotor Training is the last listed NSC study where a group of 18 participants diagnosed with complete SCI between C5 and T11 was randomized into two groups, both of which received 3–6 months of intensive locomotor training. The experimental group will receive 6.4 million UCBMNC into the dorsal root entry zones above and below the injury site. After 48 weeks, the Walking Index of Spinal Cord Injury shall be assessed, alongside Spinal Cord Independence Measure, Measure of American Spinal Injury Association Motor and Sensory Scores, and AIS. As of today, the estimated study completion date is the end of December 2024 (NCT03979742). 

For more studies to be conducted, crucial elements of safety and proper techniques for these procedure need to be further established. The aforementioned research could pave the way for future findings.

### 5.3. Hematopoietic Stem Cells

Functional neural recovery after SCI induced by HSCs’ transplantation was reported in many animal studies [87,154,178,179] There are also several clinical studies, which evaluated HSCs’ efficacy in SCI treatment [89,171,180,181,182]. In a study conducted by Deda et al., three weeks after transplantation, all of the nine patients with chronic SCI improved movements and sensations from grade A to grade B or C of the ASIA scale [89]. However, Bryukhovetskiy and Bryukhovetskiy conducted a study with 202 patients with SCI and demonstrated the quality of life improvement and restoration of movements in only 15 patients [180]. Moreover, Zakerinia et al. obtained improvement in only one of four patients with incomplete SCI and in any patient with complete SCI after HSCs’ transplantation [182]. Another clinical trial evaluated the transplantation of autologous HSCs combined with a biological scaffold containing platelet-rich plasma (PRP) for four SCI patients [171]. PRP therapy has found application in the treatment of numerous medical fields such as plastic surgery, orthopedics, dermatology, and dentistry [183,184]. The PRP is composed of trophic factors such as PDGF, VEGF, IGF, TGF-β, and bFGF [185]. Thus, the HSCs’ therapy supplemented by PRP should enhance the spinal cord regeneration through anti-inflammatory activity increased by the mentioned growth factors. However, only one patient exhibited significant motor and sensory improvement. Based on the results of the above-mentioned clinical studies, HSC transplantation shows moderate or even poor therapeutic outcomes in SCI treatment.

A recent clinical study evaluating bone marrow mononuclear cells (BMMNCs) intrathecally administered for sub-acute and chronic SCI patients at a 1.06 × 10^8^ average dose demonstrated symptomatic improvement in motor, sensory, and bladder functions without serious complications [166]. BMMNCs contain a mixture of cells including hemangioblasts, MSCs, HSCs, myeloid, lymphoid, and non-hematopoietic precursor cells [186]. Therefore, due to the combined effects of the mentioned stem cell types and satisfactory results of the above-cited study, BMMNCs represent a promising therapeutic approach for the functional recovery of patients after SCI.

## 6. Optimal Protocol for Stem Cell Administration

Among clinical studies conducted to date, there is high heterogeneity as regards dosing, transplantation phase, and route of administration. Despite that, these studies showed some improvements in clinical outcomes such as sensory scores or bladder function after stem cell therapy. However, the optimal protocol for stem cell transplantation in SCI treatment regarding its aspects discussed in this section should be investigated and implemented in further clinical studies to obtain more consistent and thus possibly better results.

### 6.1. Transplantation Route

A method of cell transplantation may be one of the major factors affecting the efficacy of stem cells for SCI treatment. For stem cell administration, four cell transplantation methods are considered—intrathecal (subarachnoid), intralesional, intravenous, and intraperitoneal routes [187].

The intralesional route is commonly used for the delivery of stem cells into the injured spinal cord in preclinical and clinical studies. A major advantage of this method is providing a maximum concentration of injected stem cells at the injury site [187,188]. However, this approach requires creating additional injury to administer cells, and in clinical practice, major surgery is necessary to expose the spinal cord appropriately [187]. Moreover, the effectiveness of this delivery method is hampered by the interaction of cells with the spinal injury microenvironment [189]. Another disadvantage of this approach is a limited amount of cells, which can be injected, because of the high risk of normal spinal cord damage due to high pressure in the injury site after injection [68]. Furthermore, this method is not recommended in patients with incomplete SCI due to the increased risk of secondary injury during surgery [170]. However, the safety of intralesional administration was demonstrated in many clinical studies [79,168,190].

The intrathecal administration is also often the chosen method in human clinical trials due to its minimal invasiveness and ease of repeatability [191]. After lumbar puncture, the injected cells reach the lesion site through cerebrospinal fluid and the so-called “homing effect”, which is a result of cells’ interaction with adhesion and chemotactic molecules such as granulocyte colony-stimulating factor (G-CSF) or calcitonin-gene related peptide (CGRP) [100,192,193]. Moreover, the intrathecal route is a more effective method for stem cell delivery compared with intravenous injection [174]. However, dispersion of cells in cerebrospinal fluid, damage of cells due to mechanical stress during injection, and impeding reaching of the injury site by adhesion of cells to the subarachnoid may reduce the therapeutic effect of stem cells delivered by intrathecal administration [187,188]. 

The intravenous route is the most commonly used in clinical trials’ method of transplantation and provides the least invasive alternative for stem cell transplantation [194]. Similarly to the homing effect after intrathecal administration, stem cells after intravenous injection migrate through the blood-spinal-cord barrier and reach the lesion site as a result of chemokines’ activity [72]. However, an intact blood-spinal-cord barrier and first-pass effect may limit reaching the lesion site by the sufficient number of stem cells [78,195]. Furthermore, this transplantation method may be related to complications such as pulmonary embolism or peripheral microthrombosis [196]. 

The use of the intraperitoneal route for stem cell delivery can prevent pulmonary embolism, which may appear after intravenous injection. Moreover, stem cells administered through the intraperitoneal route demonstrated similar outcomes compared with the intravenous route in the mice spinal cord injury model [197]. However, perforation of abdominal organs and peritonitis may occur after this route of administration. Both intravenous and intraperitoneal routes seem to be not optimal for the transplantation of stem cells in the treatment of CNS diseases such as spinal cord injury due to hindrance reaching the lesion site by stem cells and the risk of serious complications. Network meta-analysis by Chen et al. compared various delivery methods to the injured spinal cord as regards safety and treatment efficacy [173]. This study demonstrated that intrathecal administration of MSCs was associated with better outcomes in the complication rate, and ASIA motor and sensory scores compared with intralesional and intravenous routes. However, direct comparative studies are needed to establish the optimal transplantation method.

### 6.2. Timing

The timing of transplantation is another important factor determining the success of SCI therapy by stem cells. Stem cell transplantation in the acute phase of SCI is not recommended because of exposure of stem cells to the cytotoxic and ischemic environment, which is intensified in this phase of SCI and may be limited [198]. On the other hand, glial scar tissue present in the chronic phase of SCI may act as an obstacle that affects axonal regrowth [123]. However, many studies on animal models of SCI demonstrated the ability of stem cells to inhibit glial scar formation [124,199,200,201]. Based on the above findings, Oh et al. hypothesized that the subacute phase of SCI is the most appropriate period for stem cells’ transplantation [68]. A direct comparison animal study by Cheng et al. showed no significant difference in locomotor scores as regards different transplantation phases of neural stem cells, although cells administered in the subacute phase resulted in the greatest improvement [202]. A recent network meta-analysis by Shang et al. evaluating the optimal timing of neural stem cell transplantation based on animal studies also indicated the subacute phase as the best SCI phase for stem cell transplantation [203]. However, a meta-analysis based on human trials by Muthu et al. did not demonstrate significant differences [74]. Therefore, further comparative studies are necessary to clarify this aspect of stem cell therapy for SCI.

### 6.3. Dosing

The other significant aspects include the dosing and number of injections. A recent meta-analysis of clinical trials demonstrated that measured outcomes significantly improved after administration of n × 10^7^ and n × 10^8^ cell numbers [174], whereas transplanting n × 10^6^ cells was less beneficial and did not provide significant improvement [174]. Moreover, subgroup analysis of another meta-analysis demonstrated that a transplantation dose higher than 10^6^ may result in better therapeutic outcomes in comparison to lower doses [203]. The volume of injection fluid should be as low as possible because the high volume may result in secondary injury of the spinal cord [204]. Regarding the number of injections, some studies showed that multiple injections are superior compared to a single administration [68,170,205]. A study conducted by Vaquero et al. suggested that two doses of MSCs are minimum to demonstrate improvement in clinical outcomes [160]. 

## 7. Novel Therapeutic Approaches Based on Stem Cell Therapy

As it was discussed above, existing scientific data demonstrate that there are some limitations, which hamper neurological recovery of the damaged spinal cord after SCT use. Recently, researchers suggested numerous bioengineering techniques to enhance mediocre therapeutic outcomes of SCT. These novel approaches include stem-cell-derived exosomes, gene-modified stem cells, and biomaterials. (Table 3).

### 7.1. Stem-Cell-Derived Exosomes

Considering that MSCs’ secretome plays the main role in achieving therapeutic effects after MSCs’ transplantation, the use of MSCs-derived exosomes or microvesicles for SCI treatment attracted growing attention in recent years [213,214,215,216,217]. Compared with stem cell therapy, this therapeutic approach showed similar efficacy and avoids some issues such as immune rejection, dedifferentiation, a low survival rate, the risk of carcinogenicity, and difficult sourcing [208,209,210]. A recent systematic review based on animal studies demonstrated that after administration of stem-cell-derived exosomes the expression of pro-inflammatory molecules such as IL-1β and TNF-α, and apoptotic protein BAX was decreased, whereas the levels of anti-apoptotic protein Bcl-2, anti-inflammatory factors including IL-4 and IL-10 were significantly increased [218]. Moreover, the motor function was substantially enhanced. However, exosome therapy remains not fully explored and has many challenges that hamper its introduction into clinical trials such as a lack of a unified obtainment method, not entirely studying the content of exosomes, and unstandardized injection frequency, dosage, and the number of injections [206,207]. Nevertheless, the administration of MSCs-derived exosomes represents a promising alternative method for SCI treatment.

### 7.2. Gene-Modified Stem Cells 

In recent years, modifying the gene expression in controllable circumstances through genetic engineering became a potential treatment option in numerous disciplines such as oncology or regenerative medicine including therapy of SCI [21]. The knowledge about the SCI microenvironment and molecular mechanisms of action of SCT, which significantly increased during the last years, creates the opportunity to obtain desirable therapeutic outcomes through manipulating specified signaling pathways by genetically designed stem cells [219]. For example, a recent study by Huang et al. explored the safety and therapeutic outcomes of bFGF-overexpressing UCMSCs on mice models with complete SCI [122]. The bFGF-overexpressing UCMSCs complied with safety criteria for clinical application and substantially reduced glial scar formation, increased the proliferation of endogenous NSCs, enhanced neural regeneration, and improved motor recovery compared with the UCMSCs control group. The other study showed that Nogo-66 antagonistic peptide (NEP1-40)-overexpressing NSCs transplanted into enhanced axon regeneration through inhibition of the Nogo-A/NgR1 signaling pathway and remarkedly increased the differentiation capability of NSCs into neurons [220]. A recent meta-analysis based on thirty-three preclinical studies showed that animals with transplanted growth factor gene-modified cells significantly improved motor function compared with non-treated controls and animals treated with non-modified stem cells [211]. However, some major limitations exist regarding the safety of using gene-modified stem cells—the viral genome utilized for genetic modification may integrate with the host cell genome, which can result in teratoma formation [211]. Thus, in further studies, deep investigation of the safety of this method is crucial before introducing the gene-modified stem cells into human trials.

### 7.3. Biomaterials

The use of biomaterials created a new perspective for neural regeneration after SCI. Biomaterials may improve various aspects of SCI treatment by filling the cavity at the lesion site, delivering therapeutic agents, or providing a bridging role [92]. The most successful technologies utilizing biomaterials include the use of hydrogels, 3D-printed scaffolds, and nanomaterials. 

Currently, available hydrogels utilized for SCI therapy include natural hydrogels such as collagen, fibrin, fibronectin, gelatin, agarose, and alginate, as well as synthetic hydrogels including methacrylate-based hydrogels, polyethylene glycol, polylactic-co-glycolic acid, and polylactic acid [92]. A Bayesian network meta-analysis based on SCI rat models by Zhang et al. showed that the combination BMSCs with scaffolds significantly increased motor function improvement compared with scaffolds and BMSCs alone [221]. Moreover, adipose-derived stromal/stem cells (ASC), collagen, gelatin, and fibrin were demonstrated by subgroup analysis as the most effective biomaterials for scaffolds for SCI. Hydrogels may function as a carrier for the transport of cells to the lesion site, may produce bioactive molecules protecting transplanted cells against the SCI microenvironment, or can enhance migration, proliferation, and differentiation of administered stem cells by providing 3D support [92]. A recent animal study demonstrated increased neuroprotection and immunomodulation after combined transplantation of MSCs and a nanofiber-hydrogel composite compared with MSCs or the nanofiber-hydrogel composite alone [222]. However, there are some limitations of hydrogel application such as the degradation rate, low durability, decreased mechanical strength, and concerns with stem cell loading into hydrogels followed by releasing them at a defined stage of SCI [92]. Nevertheless, a clinical trial investigating collagen NeuroRegen scaffolds combined with UCMSCs was conducted with no adverse effects and good results including recovery of sensory and motor functions [169].

Recently, 3D bioprinting technology was significantly popularized in numerous fields of medicine including regenerative medicine [223,224,225]. 3D-bioprinted scaffolds are designed to protect the stem cells against the inflammatory environment and enhance their differentiation and integration at the lesion site [226]. For the use for the treatment of SCI, two categories of 3D bioprinting can be divided such as acellular biomaterial 3D printing and 3D bioprinting loaded with cells or factors. Numerous preclinical studies demonstrated significant improvements in neurological recovery after transplantation of 3D-bioprinted scaffolds manufactured from collagen/silk fibroin, collagen/heparin, collagen/chitosan, or alginate [227,228,229]. Moreover, during the fabrication of acellular 3D-printed scaffolds, toxic cross-linking reagents, high temperatures, and UV radiation may be used in contrast to 3D bioprinting of loaded cells [212]. Due to the use of bioinkswith added MSCs or NSCs, 3D bioprinting enables the creation of a “spinal cord-like” scaffold containing a high number of stem cells designed for axon reconnection [212]. These constructs improved neural regeneration and formed new neural pathways across the lesion site in animal models [230]. Both acellular and cell-loaded 3D-bioprinted scaffolds constitute a promising therapeutic approach for neural regeneration after SCI. However, the risk of immune rejection, a limited number of printable bioinks, unadapted mechanical properties to natural tissues, and troublesome bioprinting procedures are relevant concerns hampering the therapeutic capacity of 3D-bioprinted scaffolds [212]. 

The nanomaterials may be used as nano-carriers for drug delivery into the lesion site, e.g., methylprednisolone [231]. Moreover, combined with stem cell transplantation, the use of magnetic nanoparticles may improve stem cell transport after administration and increase their viability at the lesion site [92]. However, the systemic clearance of nanomaterials and the effects of their degradation products on human organisms are insufficiently studied [92]. Nevertheless, a combined therapeutic approach utilizing nanomaterials and stem cells constitutes promising utility for better neural regeneration after SCI. 

## 8. Challenges, Barriers, and Future Directions

In recent years, numerous clinical trials evaluating MSCs, NSCs, and HSCs were conducted with varied results demonstrating mainly mild improvement of motor, sensory, and urinary functions without serious complications. However, mean follow-up time, transplantation route, dose, and timing of stem cell administration significantly vary in the available clinical trials, which complicates comparing their results and establishing the consensus regarding the optimal protocol of stem cell administration and follow-up duration. Moreover, none of the conducted clinical trials to date demonstrated significant improvement in motor function in SCI patients after stem cell administration alone. Thus, the clinical outcomes of stem cell therapy seem to be mediocre. Furthermore, gunshot wounds were excluded from discussed clinical trials. So far, no clinical trial was conducted on this topic [232]. Hence, it may be valuable to investigate this aspect of stem cell therapy in further clinical research. Future studies should also consider evaluating the combination of the stem cell therapy with various types of rehabilitation training including locomotor training, robotic-assisted treadmill training, or epidural spinal cord stimulation [233,234,235].

Recent studies are looking for therapeutic approaches, which may improve the treatment efficacy of stem cell therapy. The growing knowledge about SCI pathophysiology and molecular pathways involved in neural regeneration creates an opportunity to develop novel treatment modalities based on stem cells. Indeed, evidence from recent experimental research provided emerging strategies such as stimulation of macrophage polarization from M1 into the M2 phenotype through miRNAs or anti-inflammatory drugs. [236,237]. Moreover, as discussed in the former section, methods such as gene-modified stem cells, stem cell-derived exosomes, and stem cell transplantation combined with the use of collagen scaffolds represent promising techniques for enhancing the improvement of neural function and even restoration of damaged neural pathways in the injured spinal cord.

## 9. Conclusions

The therapy using multipotent stem cells for SCI demonstrated a high potential for promoting neural recovery after spinal injuries. However, their clinical efficacy was questioned by current clinical evidence. There are numerous challenges that researchers should overcome to increase the effectiveness of stem cell therapy, such as stem cell immunogenicity, lack of stem cell differentiation in the SCI microenvironment, and obtaining the optimal administration protocol.

Moreover, multiple factors in the SCI microenvironment are responsible for failed neural recovery. Furthermore, SCI pathophysiology remains not thoroughly investigated. A deep understanding of molecular interactions between transplanted stem cells and the SCI microenvironment appears to be crucial for the therapeutic success of stem cell therapy. However, a therapy that utilizes only stem cell transplantation is insufficient to provide successful neural recovery after SCI. Hence, combinatorial therapies seem to be the most promising therapeutic approaches. 

Finally, we hope that appropriately modified stem cell therapy, therapies based on stem cell therapy, or combinatorial approaches with other treatment methods may further improve neural regeneration of damaged spinal cord structures and contribute to more effective treatment of patients with this devastating condition.

## Figures and Tables

**Table 1:** Main characteristics of various stem cell types investigated for application in Spinal Cord Injury treatment.

Type of Stem Cells	Differentiation Potential	Sourcing	Main Advantages	Limitations	Application in Spinal Cord Injury	Refs
Embryonal Stem Cells	totipotent, pluripotent	morula, blastocyst, umbilical cord, amniotic fluid, amnion, chorion, generated from adult somatic cells	possibility to generate any cell lines, e.g., neurons or oligodendrocytes	the risk of immune rejection, the ethical concern regarding the use of human embryos, the risk of tumorigenicity	Preclinical studies	[16,49,50,52,56,61]
Induced Pluripotent Stem Cells	pluripotent	generated from adult somatic cells using so-called OSKM transcription factors	lack of ethical issues and immune suppression (in autologous method)	the risk of immune rejections, instability of iPSCs’ genome, potential tumorigenicity	Preclinical studies	[52,64,65,66,92]
Mesenchymal Stem Cells	multipotent	bone marrow, umbilical cord blood, adipose tissue	capability to generate adipocytes, bone, and chondrocytes, easy extraction, rapid proliferation, low immunogenicity; ADMSCs and BMSCs can be generated without ethical issues	ADMSCs and BMSCs require liposuction or bone marrow aspirate followed by cultivation, which makes them time-consuming, and expensive sources; Umbilical cord or Wharton’s Jelly MSCs require conducting complex procedures namely lyophilization to avoid immunological responses and are controversial from the ethical point of view	Clinical studies	[14,27,53,68,69,70,73,74,76]
Hematopoietic Stem Cells	multipotent	placenta, cord blood, adult bone marrow	capability to differentiate into all cell types of the hematopoietic system, treatment for many diseases such as hematopoietic diseases, multiple sclerosis, Cron’s disease, and diabetes	the risk of immune rejection	Clinical studies	[53,61,84,85,86]
Neural Stem Cells	multipotent	ventricular system of the brain, central canal of the spinal cord, dentate gyrus of the hippocampus, differentiation from somatic cells, iPSCs	capability to differentiate into neurons, oligodentrocytes and astrocytes	the risk of immune rejection, low progress of the research due to ethical and financial problems	Clinical studies	[92]

**Table 2:** Recent clinical studies investigating Stem Cells Therapy for Spinal Cord Injury from the years 2017–2022.

Clinical Study	Type of Stem Cells	Study Design	Phase of Study	Country	Number of Patients	The Initial ASIA Grade	The Initial Phase of SCI	Route of Administration	Dose of Cells	Combined with	Follow-Up Duration	Clinical Outcomes	Adverse Effects
Saini et al. 2022 [27]	BMSCs	randomized placebo controlled trial	II	India	27	A	acute	intramedullary	2 × 10^8^	n/a	3 years	improvement in 6 patients of stem cells group and 1 in placebo group in ASIA score	n/a
Zamani et al. 2022 [163]	BMSCs	non-randomized open-labeled controlled trial	I	Iran	3	A	chronic	subarachnoid	3 × 10^7^	OECs	2 years	1 patient improved from A to B in AISA score	no significant adverse effects, mostly headache and neurophatic pain
Smirnov et al. 2022 [32]	UCBCs	non-randomized open-labeled controlled trial	I/II	Russian Federation	10	A (n = 6), B (n = 4)	acute	intravenous	4 doses, 1.2 × 10^9^	n/a	12 months	the mean increase in level of ASIA was 2.2 points; the 1-year LEMS parameter was >25 points in 6 patients	no significant adverse effects
Albu et al. 2021 [14]	WJ-MSCs	randomized placebo controlled trial	I/II	Spain	10	A	chronic	subarachnoid	1 × 10^7^	n/a	6 months	improvement sensation in the dermatomes below the level of injury in stem cells group; decrease neurogenic hyperactivity in bladder, decrease external sphincter dyssynergy, increase maximum capacity and compliance in bladder	no significant adverse effects
Yang et al. 2021 [28]	UCMSCs	prospective single-arm study	I/II	China	41	A, B, C, D	chronic	subarachnoid	4 doses, 1 × 10^6^ cells/kg	n/a	12 months	ASIA and IANR-SCIFRS total scores revealed statistical increases, mainly reflected in the improvement of pinprick, light touch, motor and sphincter scores, decrease in muscle spasticity	no significant side effects, mostly fever and headache
Oraee-Yazdani et al. 2021 [164]	BMSCs	single-arm study	I/II	Iran	11	A	subacute	subarachnoid	3 × 10^8^	Schwann cells	12 months	positive sensory changes in AIS score, motor recovery; improvement in the trunk movement, equilibrium in standing/sitting positions, a reduction in the severity of constipation, improvement in sensation of the filling bladder and rectum, empowerment of voiding	increase in spasticity, numbness, or tingling sensation, neuropathic pain, headache and facial flushing
Deng et al 2020 [165]	UC-MSCs	non-randomized open-labeled controlled trial	I	China	40	A	acute	intramedullary	4 × 10^7^	collagen scaffolds	12 months	improvement in urinary functions and ASIA score in treatment group	no significant adverse effects
Curt et al. 2020 [30]	CNS- NSCs	non-randomized open-labeled controlled trial	I/II	Switzerland, Canada	12	A (n = 7), B (n = 5)	chronic	intramedullary	2 × 10^7^	n/a	6 years	improvement with reliable sensory improvements	headache, spasticity, pressure ulcer, erythema
Sharma et al. 2020 [166]	BMMNCs	non-randomized open-labeled controlled trial	II	India	180	A (n = 138), B (n = 28), C (n = 10), D (n = 3)	subacute and acute	subarachnoid	1.06 × 10^8^	n/a	9 ± 7 months	statistically significant improvement on FIM and WISCI scores	no significant adverse effects, mostly fever, headache
Levi et al. 2019 [31]	CNS- NSCs	randomized single-blinded controlled trial	II	United States	16	A (n = 3), B (n = 9)	chronic	intramedullary	1.5 × 10^7^–4 × 10^7^	n/a	12 months	no significant improvement	musculoskeletal pain and infections
Levi et al. 2018 [167]	CNS- NSCs	non-randomized open-labeled controlled trial	I/II	United States	12	A (n = 8), B (n = 4)	chronic	intramedullary	2 × 10^7^	n/a	28–57 months	n/a	cerebrospinal fluid leakage, constipation and UTI, staph epidermidis wound infection, autonomic dysreflexia, postprocedural sepsis, posterior reversible encephalopathy syndrome, constipation, seizure, wound hematoma, aphasia
Levi et al. 2018 [167]	CNS- NSCs	randomized single-blinded controlled trial	II	United States	17	A (n = 3), B (n = 14)	chronic	intramedullary	1.5 × 10^7^–4 × 10^7^	n/a	1–12 months		
Curtis et al. 2018 [168]	SC- NSCs	single-arm study	I	United States	4	A	chronic	intramedullary	1.2 × 10^6^	n/a	60 months	no significant improvement	no adverse effects
Xiao et al. 2018 [169]	UCMSCs	single-arm study	I	China	2	A	acute	intramedullary	4 × 10^7^	collagen scaffolds	1 year	recovery of the sensory and motor functions; the sensory level expanded below the injury level, and the patients regained the sense function in bowel and bladder; 2 patients were improved from ASIA A to ASIA C; the recovery of the interrupted neural conduction	no adverse effects
Vaquero et al. 2018 [160]	BMSCs	non-randomized open-labeled uncontrolled trial	II	Spain	11	A (n = 3), B (n = 4), C (n = 3), D (n = 1)	chronic	subarachnoid	1 × 10^8^	n/a	10 months	improvement in sensitivity, motor power, spasms, spasticity, neuropathic pain, sexual function or sphincter dysfunction; 3 patients, initially classified as ASIA A, B and C, changed to ASIA B, C and D; decrease in postmicturition residue and improvement in bladder compliance; improvement in somatosensory or motor-evoked potentials, improvement in voluntary muscle contraction together with infralesional active muscle reinnervation	no significant adverse effects, mostly transitory sciatic pain, headaches, pain in the area of lumbar puncture
Vaquero et al. 2017 [170]	BMSCs	non-randomized open-labeled uncontrolled trial	I	Spain	10	B (n = 4), C (n = 5), D (n = 1)	chronic	subarachnoid	4 doses, 3 × 10^7^	n/a	12 months	improvement in sensitivity and motor function; improvement of sexual function; neuropathic pain disappeared or decreased; improvement in bladder and bowel control; improvement in spasms; decrease in spasticity	no significant adverse effects, mostly headaches and pain in the area of lumbar puncture
Ammar et al. 2017 [171]	HSCs	single-arm study	I	Saudi Arabia	4	A	chronic	intramedullary	2.8 × 10^6^	PRP	2–3 years	One patient demonstrated motor and objective sensory improvement (P = 0.05); two other patients reported subjective sensory improvement, and the fourth one remained without any improvement	no adverse effects

*Abbreviations:* BMSCs—bone-marrow mesenchymal stem cells; UCBCs—umbilical cord blood cells; WJ-MSCs—Wharton jelly mesenchymal stem cells; CNS-NSCs—central nervous system neural stem cells; SC-NSCs—spinal cord neural stem cells; UCMSCs—umblilical cord mesenchymal stem cells; BMMNCs—bone marrow mononuclear cells; OECs—olfactory ensheathing cells; ASIA—American Spine Injury Association; RCT—randomized controlled trial; SCI—spinal cord injury; PRP—platelet-rich plasma; n/a—non-applicable/not available.

**Table 3:** Emerging therapies based on Stem Cell Therapy.

Technology		Phase of Studies	Advantages	Limitations	Refs
Stem cell-derived exosomes		preclinical	comparable effectiveness with SCT avoids immune rejection and risk of carcinogenicity, avoids problems with low survival rate, dedifferentiation, and difficult obtainment of stem cells	not entirely studied the content of exosomes, lack of unified obtainment procedure, unstandardized number of injections, its frequency, and dosage	[206,207,208,209,210]
Gene-modified stem cells		preclinical	better outcomes compared with non-modified stem cells, enables manipulation of the specific molecular pathways of spinal cord injury microenvironment to enhance treatment efficacy	safety concerns regarding the use of viral vectors for genetic engineering	[211]
Biomaterials	Cell-free 3D-printed scaffolds	preclinical	creates a suitable microenvironment for stem cells, provides a bridging role, improves neural regeneration, resistance to toxic, temperature, and UV radiation during the fabrication process	immune rejection, cumbersome bioprinting procedure, limited availability of printable bioinks	[92,212]
	3D-printed scaffold loaded with stem cells	preclinical	possibility to create a "spinal cord-like" scaffold	restricted conditions of the manufacturing process, immune rejection, cumbersome bioprinting procedure, limited availability of printable bioinks	[92,212]
	Hydrogels	clinical	high biocompatibility may be used as a cell or cell factors’ carrier for its transport into the lesion site	fast degradation rate, low mechanical strength, and durability	[92]
	Nanomaterials	preclinical	improves stem cell transport and viability	not established release time and dose of drugs loaded on nanoparticles	[92]

## Data Availability

Not applicable.

## References

[1] Lo J., Chan L., Flynn S. (2021). A Systematic Review of the Incidence, Prevalence, Costs, and Activity/Work Limitations of Amputation, Osteoarthritis, Rheumatoid Arthritis, Back Pain, Multiple Sclerosis, Spinal Cord Injury, Stroke, and Traumatic Brain Injury in the United States: A 2019 Update. Arch. Phys. Med. Rehabil..

[2] Chay W., Kirshblum S. (2020). Predicting Outcomes After Spinal Cord Injury. Phys. Med. Rehabil. Clin. N. Am..

[3] Michel M., Goldman M., Peart R., Martinez M., Reddy R., Lucke-Wold B. (2021). Spinal Cord Injury: A Review of Current Management Considerations and Emerging Treatments. J. Neurol. Sci. Res..

[4] Haddad A.F., Burke J.F., Dhall S.S. (2021). The Natural History of Spinal Cord Injury. Neurosurg. Clin. N. Am..

[5] Golestani A., Shobeiri P., Sadeghi-Naini M., Jazayeri S.B., Maroufi S.F., Ghodsi Z., Dabbagh Ohadi M.A., Mohammadi E., Rahimi-Movaghar V., Ghodsi S.M. (2022). Epidemiology of Traumatic Spinal Cord Injury in Developing Countries from 2009 to 2020: A Systematic Review and Meta-Analysis. Neuroepidemiology.

[6] Kim G.-U., Sung S.-E., Kang K.-K., Choi J.-H., Lee S., Sung M., Yang S.Y., Kim S.-K., Kim Y.I., Lim J.-H. (2021). Therapeutic Potential of Mesenchymal Stem Cells (MSCs) and MSC-Derived Extracellular Vesicles for the Treatment of Spinal Cord Injury. Int. J. Mol. Sci..

[7] Smith É., Fitzpatrick P., Lyons F., Morris S., Synnott K. (2022). Epidemiology of Non-Traumatic Spinal Cord Injury in Ireland—A Prospective Population-Based Study. J. Spinal Cord Med..

[8] Litak J., Czyżewski W., Szymoniuk M., Sakwa L., Pasierb B., Litak J., Hoffman Z., Kamieniak P., Roliński J. (2022). Biological and Clinical Aspects of Metastatic Spinal Tumors. Cancers.

[9] Gober J., Thomas S.P., Gater D.R. (2022). Pediatric Spina Bifida and Spinal Cord Injury. J. Pers. Med..

[10] Sahbani K., Cardozo C.P., Bauman W.A., Tawfeek H.A. (2022). Inhibition of TGF-Beta Signaling Attenuates Disuse-Induced Trabecular Bone Loss After Spinal Cord Injury in Male Mice. Endocrinology.

[11] Fehlings M.G., Tetreault L.A., Wilson J.R., Aarabi B., Anderson P., Arnold P.M., Brodke D.S., Burns A.S., Chiba K., Dettori J.R. (2017). A Clinical Practice Guideline for the Management of Patients with Acute Spinal Cord Injury and Central Cord Syndrome: Recommendations on the Timing (≤24 Hours Versus >24 Hours) of Decompressive Surgery. Glob. Spine J..

[12] Fehlings M.G., Wilson J.R., Tetreault L.A., Aarabi B., Anderson P., Arnold P.M., Brodke D.S., Burns A.S., Chiba K., Dettori J.R. (2017). A Clinical Practice Guideline for the Management of Patients with Acute Spinal Cord Injury: Recommendations on the Use of Methylprednisolone Sodium Succinate. Glob. Spine J..

[13] Wang T.Y., Park C., Zhang H., Rahimpour S., Murphy K.R., Goodwin C.R., Karikari I.O., Than K.D., Shaffrey C.I., Foster N. (2021). Management of Acute Traumatic Spinal Cord Injury: A Review of the Literature. Front. Surg..

[14] Albu S., Kumru H., Coll R., Vives J., Vallés M., Benito-Penalva J., Rodríguez L., Codinach M., Hernández J., Navarro X. (2021). Clinical Effects of Intrathecal Administration of Expanded Wharton Jelly Mesenchymal Stromal Cells in Patients with Chronic Complete Spinal Cord Injury: A Randomized Controlled Study. Cytotherapy.

[15] Costăchescu B., Niculescu A.G., Dabija M.G., Teleanu R.I., Grumezescu A.M., Eva L. (2022). Novel Strategies for Spinal Cord Regeneration. Int. J. Mol. Sci..

[16] Hoang D.M., Pham P.T., Bach T.Q., Ngo A.T.L., Nguyen Q.T., Phan T.T.K., Nguyen G.H., Le P.T.T., Hoang V.T., Forsyth N.R. (2022). Stem Cell-Based Therapy for Human Diseases. Signal Transduct. Target. Ther..

[17] Pucułek M., Baj J., Portincasa P., Sitarz M., Grochowski C., Radzikowska E. (2021). The Morphology and Application of Stem Cells in Digestive System Surgery. Folia Morphol..

[18] Li T.-T., Wang Z.-R., Yao W.-Q., Linghu E.-Q., Wang F.-S., Shi L. (2022). Stem Cell Therapies for Chronic Liver Diseases: Progress and Challenges. Stem Cells Transl. Med..

[19] Gruca D., Zając M., Wróblewski W., Borowiecka M., Buksak D. (2022). The Relation between Adipose-Derived Stem Cells and Wound Healing Process—The Review. J. Educ. Health Sport.

[20] Sarsenova M., Issabekova A., Abisheva S., Rutskaya-Moroshan K., Ogay V., Saparov A. (2021). Mesenchymal Stem Cell-Based Therapy for Rheumatoid Arthritis. Int. J. Mol. Sci..

[21] Hassanzadeh A., Shamlou S., Yousefi N., Nikoo M., Verdi J. (2022). Genetically-Modified Stem Cell in Regenerative Medicine and Cancer Therapy; A New Era. Curr. Gene Ther..

[22] Puranik N., Arukha A.P., Yadav S.K., Yadav D., Jin J.O. (2022). Exploring the Role of Stem Cell Therapy in Treating Neurodegenerative Diseases: Challenges and Current Perspectives. Curr. Stem Cell Res. Ther..

[23] Ejma M., Madetko N., Brzecka A., Alster P., Budrewicz S., Koszewicz M., Misiuk-Hojło M., Tomilova I.K., Somasundaram S.G., Kirkland C.E. (2022). The Role of Stem Cells in the Therapy of Stroke. Curr. Neuropharmacol..

[24] Peterson S., Jalil A., Beard K., Kakara M., Sriwastava S. (2022). Updates on Efficacy and Safety Outcomes of New and Emerging Disease Modifying Therapies and Stem Cell Therapy for Multiple Sclerosis: A Review. Mult. Scler. Relat. Disord..

[25] Li X., Sundström E. (2022). Stem Cell Therapies for Central Nervous System Trauma: The 4 Ws—What, When, Where, and Why. Stem Cells Transl. Med..

[26] Zipser C.M., Cragg J.J., Guest J.D., Fehlings M.G., Jutzeler C.R., Anderson A.J., Curt A. (2022). Cell-Based and Stem-Cell-Based Treatments for Spinal Cord Injury: Evidence from Clinical Trials. Lancet Neurol..

[27] Saini R., Pahwa B., Agrawal D., Singh P.K., Gujjar H., Mishra S., Jagdevan A., Misra M.C. (2022). Efficacy and Outcome of Bone Marrow Derived Stem Cells Transplanted via Intramedullary Route in Acute Complete Spinal Cord Injury—A Randomized Placebo Controlled Trial. J. Clin. Neurosci..

[28] Yang Y., Pang M., Du C., Liu Z.-Y., Chen Z.-H., Wang N.-X., Zhang L.-M., Chen Y.-Y., Mo J., Dong J.-W. (2021). Repeated Subarachnoid Administrations of Allogeneic Human Umbilical Cord Mesenchymal Stem Cells for Spinal Cord Injury: A Phase 1/2 Pilot Study. Cytotherapy.

[29] Hur J.W., Cho T.H., Park D.H., Lee J.B., Park J.Y., Chung Y.G. (2016). Intrathecal Transplantation of Autologous Adipose-Derived Mesenchymal Stem Cells for Treating Spinal Cord Injury: A Human Trial. J. Spinal Cord Med..

[30] Curt A., Hsieh J., Schubert M., Hupp M., Friedl S., Freund P., Huber E., Pfyffer D., Sutter R., Jutzeler C. (2020). The Damaged Spinal Cord Is a Suitable Target for Stem Cell Transplantation. Neurorehabil. Neural Repair.

[31] Levi A.D., Anderson K.D., Okonkwo D.O., Park P., Bryce T.N., Kurpad S.N., Aarabi B., Hsieh J., Gant K. (2019). Clinical Outcomes from a Multi-Center Study of Human Neural Stem Cell Transplantation in Chronic Cervical Spinal Cord Injury. J. Neurotrauma.

[32] Smirnov V.A., Radaev S.M., Morozova Y.V., Ryabov S.I., Yadgarov M.Y., Bazanovich S.A., Lvov I.S., Talypov A.E., Grin’ A.A. (2022). Systemic Administration of Allogeneic Cord Blood Mononuclear Cells in Adults with Severe Acute Contusion Spinal Cord Injury: Phase 1/2a Pilot Clinical Study-Safety and Primary Efficacy Evaluation. World Neurosurg..

[33] Gant K.L., Guest J.D., Palermo A.E., Vedantam A., Jimsheleishvili G., Bunge M.B., Brooks A.E., Anderson K.D., Thomas C.K., Santamaria A.J. (2022). Phase 1 Safety Trial of Autologous Human Schwann Cell Transplantation in Chronic Spinal Cord Injury. J. Neurotrauma.

[34] Tabakow P., Jarmundowicz W., Czapiga B., Fortuna W., Miedzybrodzki R., Czyz M., Huber J., Szarek D., Okurowski S., Szewczyk P. (2013). Transplantation of Autologous Olfactory Ensheathing Cells in Complete Human Spinal Cord Injury. Cell Transplant..

[35] Rövekamp M., von Glinski A., Volkenstein S., Dazert S., Sengstock C., Schildhauer T.A., Breisch M. (2022). Olfactory Stem Cells for the Treatment of Spinal Cord Injury-A New Pathway to the Cure?. World Neurosurg..

[36] Fessler R.G., Ehsanian R., Liu C.Y., Steinberg G.K., Jones L., Lebkowski J.S., Wirth E.D., McKenna S.L. (2022). A Phase 1/2a Dose-Escalation Study of Oligodendrocyte Progenitor Cells in Individuals with Subacute Cervical Spinal Cord Injury. J. Neurosurg. Spine.

[37] Liu J., Gao J., Liang Z., Gao C., Niu Q., Wu F., Zhang L. (2022). Mesenchymal Stem Cells and Their Microenvironment. Stem Cell Res. Ther..

[38] Ahuja C.S., Nori S., Tetreault L., Wilson J., Kwon B., Harrop J., Choi D., Fehlings M.G. (2017). Traumatic Spinal Cord Injury—Repair and Regeneration. Clin. Neurosurg..

[39] Garcia E., Aguilar-Cevallos J., Silva-Garcia R., Ibarra A. (2016). Cytokine and Growth Factor Activation in Vivo and in Vitro after Spinal Cord Injury. Mediat. Inflamm..

[40] Zhao Q., Liu F., Zhou B., Liu H., Wang X., Li S. (2022). Ferroptosis: A Novel Therapeutic Direction of Spinal Cord Injury. Comput. Math. Methods Med..

[41] Slater P.G., Domínguez-Romero M.E., Villarreal M., Eisner V., Larraín J. (2022). Mitochondrial Function in Spinal Cord Injury and Regeneration. Cell. Mol. Life Sci..

[42] Salman M.M., Kitchen P., Halsey A., Wang M.X., Törnroth-Horsefield S., Conner A.C., Badaut J., Iliff J.J., Bill R.M. (2022). Emerging Roles for Dynamic Aquaporin-4 Subcellular Relocalization in CNS Water Homeostasis. Brain.

[43] Fan B., Wei Z., Yao X., Shi G., Cheng X., Zhou X., Zhou H., Ning G., Kong X., Feng S. (2018). Microenvironment Imbalance of Spinal Cord Injury. Cell Transplant..

[44] Beirowski B., Nógrádi A., Babetto E., Garcia-Alias G., Coleman M.P. (2010). Mechanisms of Axonal Spheroid Formation in Central Nervous System Wallerian Degeneration. J. Neuropathol. Exp. Neurol..

[45] Alizadeh A., Dyck S.M., Karimi-Abdolrezaee S. (2019). Traumatic Spinal Cord Injury: An Overview of Pathophysiology, Models and Acute Injury Mechanisms. Front. Neurol..

[46] Jia M., Njapo S.A.N., Rastogi V., Hedna V.S. (2015). Taming Glutamate Excitotoxicity: Strategic Pathway Modulation for Neuroprotection. CNS Drugs.

[47] Brat D.J. (2018). Normal Brain Histopathology. Practical Surgical Neuropathology: A Diagnostic Approach.

[48] Sharma K., Zhang G., Li S. (2015). Astrogliosis and Axonal Regeneration. Neural Regen..

[49] Bacakova L., Zarubova J., Travnickova M., Musilkova J., Pajorova J., Slepicka P., Kasalkova N.S., Svorcik V., Kolska Z., Motarjemi H. (2018). Stem Cells: Their Source, Potency and Use in Regenerative Therapies with Focus on Adipose-Derived Stem Cells—A Review. Biotechnol. Adv..

[50] Sobhani A., Sobhani A., Khanlarkhani N., Baazm M., Mohammadzadeh F., Najafi A., Mehdinejadiani S., Aval F.S. (2017). Multipotent Stem Cell and Current Application. Acta Med. Iran..

[51] Zakrzewski W., Dobrzyński M., Szymonowicz M., Rybak Z. (2019). Stem Cells: Past, Present, and Future. Stem Cell Res. Ther..

[52] Takahashi K., Tanabe K., Ohnuki M., Narita M., Ichisaka T., Tomoda K., Yamanaka S. (2007). Induction of Pluripotent Stem Cells from Adult Human Fibroblasts by Defined Factors. Cell.

[53] Dulak J., Szade K., Szade A., Nowak W., Józkowicz A. (2015). Adult Stem Cells: Hopes and Hypes of Regenerative Medicine* Definition of Stem and Progenitor Cell. Acta Biochim. Pol..

[54] Mirzaei H., Sahebkar A., Sichani L.S., Moridikia A., Nazari S., Nahand J.S., Salehi H., Stenvang J., Masoudifar A., Mirzaei H.R. (2018). Therapeutic Application of Multipotent Stem Cells. J. Cell. Physiol..

[55] Paliwal S., Fiumera H.L., Mohanty S. (2021). Stem Cell Plasticity and Regenerative Potential Regulation through Ca^2+^-Mediated Mitochondrial Nuclear Crosstalk. Mitochondrion.

[56] Grochowski C., Radzikowska E., Maciejewski R. (2018). Neural Stem Cell Therapy—Brief Review. Clin. Neurol. Neurosurg..

[57] Hawkins K.E., Corcelli M., Dowding K., Ranzoni A.M., Vlahova F., Hau K.L., Hunjan A., Peebles D., Gressens P., Hagberg H. (2018). Embryonic Stem Cell-Derived Mesenchymal Stem Cells (MSCs) Have a Superior Neuroprotective Capacity Over Fetal MSCs in the Hypoxic-Ischemic Mouse Brain. Stem Cells Transl. Med..

[58] Xia Y., Hu G., Chen Y., Yuan J., Zhang J., Wang S., Li Q., Wang Y., Deng Z. (2021). Embryonic Stem Cell Derived Small Extracellular Vesicles Modulate Regulatory T Cells to Protect against Ischemic Stroke. ACS Nano.

[59] Wang X., Kimbrel E.A., Ijichi K., Paul D., Lazorchak A.S., Chu J., Kouris N.A., Yavanian G.J., Lu S.J., Pachter J.S. (2021). Human ESC-Derived MSCs Outperform Bone Marrow MSCs in the Treatment of an EAE Model of Multiple Sclerosis. Stem Cell Rep..

[60] Araki R., Mizutani E., Hoki Y., Sunayama M., Wakayama S., Nagatomo H., Kasama Y., Nakamura M., Wakayama T., Abe M. (2017). The Number of Point Mutations in Induced Pluripotent Stem Cells and Nuclear Transfer Embryonic Stem Cells Depends on the Method and Somatic Cell Type Used for Their Generation. Stem Cells.

[61] Mousaei Ghasroldasht M., Seok J., Park H.S., Liakath Ali F.B., Al-Hendy A. (2022). Stem Cell Therapy: From Idea to Clinical Practice. Int. J. Mol. Sci..

[62] Poetsch M.S., Strano A., Guan K. (2022). Human Induced Pluripotent Stem Cells: From Cell Origin, Genomic Stability, and Epigenetic Memory to Translational Medicine. Stem Cells.

[63] Du X., Amponsah A.E., Kong D., He J., Ma Z., Ma J., Cui H. (2022). HiPSC-Neural Stem/Progenitor Cell Transplantation Therapy for Spinal Cord Injury. Curr. Stem Cell Res. Ther..

[64] Ji P., Manupipatpong S., Xie N., Li Y. (2016). Induced Pluripotent Stem Cells: Generation Strategy and Epigenetic Mystery behind Reprogramming. Stem Cells Int..

[65] Fu X. (2014). The Immunogenicity of Cells Derived from Induced Pluripotent Stem Cells. Cell. Mol. Immunol..

[66] Lee A.S., Tang C., Rao M.S., Weissman I.L., Wu J.C. (2013). Tumorigenicity as a Clinical Hurdle for Pluripotent Stem Cell Therapies. Nat. Med..

[67] Attia N., Mashal M. (2021). Mesenchymal Stem Cells: The Past Present and Future. Adv. Exp. Med. Biol..

[68] Oh S.K., Choi K.H., Yoo J.Y., Kim D.Y., Kim S.J., Jeon S.R. (2016). A Phase III Clinical Trial Showing Limited Efficacy of Autologous Mesenchymal Stem Cell Therapy for Spinal Cord Injury. Neurosurgery.

[69] Vaquero J., Zurita M., Rico M.A., Aguayo C., Fernandez C., Rodriguez-Boto G., Marin E., Tapiador N., Sevilla M., Carballido J. (2018). Cell Therapy with Autologous Mesenchymal Stromal Cells in Post-Traumatic Syringomyelia. Cytotherapy.

[70] Peng C., Li Y., Lu L., Zhu J., Li H., Hu J. (2019). Efficient One-Step Induction of Human Umbilical Cord-Derived Mesenchymal Stem Cells (UC-MSCs) Produces MSC-Derived Neurospheres (MSC-NS) with Unique Transcriptional Profile and Enhanced Neurogenic and Angiogenic Secretomes. Stem Cells Int..

[71] Qu J., Zhang H. (2017). Roles of Mesenchymal Stem Cells in Spinal Cord Injury. Stem Cells Int..

[72] Cofano F., Boido M., Monticelli M., Zenga F., Ducati A., Vercelli A., Garbossa D. (2019). Mesenchymal Stem Cells for Spinal Cord Injury: Current Options, Limitations, and Future of Cell Therapy. Int. J. Mol. Sci..

[73] Jiang W., Xu J. (2020). Immune Modulation by Mesenchymal Stem Cells. Cell Prolif..

[74] Muthu S., Jeyaraman M., Gulati A., Arora A. (2021). Current Evidence on Mesenchymal Stem Cell Therapy for Traumatic Spinal Cord Injury: Systematic Review and Meta-Analysis. Cytotherapy.

[75] Han S., Sun H.M., Hwang K.C., Kim S.W. (2015). Adipose-Derived Stromal Vascular Fraction Cells: Update on Clinical Utility and Efficacy. Crit. Rev. Eukaryot. Gene Expr..

[76] Berebichez-Fridman R., Montero-Olvera P.R. (2018). Sources and Clinical Applications of Mesenchymal Stem Cells: State-of-the-Art Review. Sultan Qaboos Univ. Med. J..

[77] Marino L., Castaldi M.A., Rosamilio R., Ragni E., Vitolo R., Fulgione C., Castaldi S.G., Serio B., Bianco R., Guida M. (2019). Mesenchymal Stem Cells from the Wharton’s Jelly of the Human Umbilical Cord: Biological Properties and Therapeutic Potential. Int. J. Stem Cells.

[78] Liau L.L., Looi Q.H., Chia W.C., Subramaniam T., Ng M.H., Law J.X. (2020). Treatment of Spinal Cord Injury with Mesenchymal Stem Cells. Cell Biosci..

[79] Anderson K.D., Guest J.D., Dietrich W.D., Bartlett Bunge M., Curiel R., Dididze M., Green B.A., Khan A., Pearse D.D., Saraf-Lavi E. (2017). Safety of Autologous Human Schwann Cell Transplantation in Subacute Thoracic Spinal Cord Injury. J. Neurotrauma.

[80] Andreopoulou E., Arampatzis A., Patsoni M., Kazanis I. (2017). Being a Neural Stem Cell: A Matter of Character but Defined by the Microenvironment. Adv. Exp. Med. Biol..

[81] Shahbazi E., Moradi S., Nemati S., Satarian L., Basiri M., Gourabi H., Zare Mehrjardi N., Günther P., Lampert A., Händler K. (2016). Conversion of Human Fibroblasts to Stably Self-Renewing Neural Stem Cells with a Single Zinc-Finger Transcription Factor. Stem Cell Rep..

[82] García-González D., Murcia-Belmonte V., Esteban P.F., Ortega F., Díaz D., Sánchez-Vera I., Lebrón-Galán R., Escobar-Castañondo L., Martínez-Millán L., Weruaga E. (2016). Anosmin-1 over-Expression Increases Adult Neurogenesis in the Subventricular Zone and Neuroblast Migration to the Olfactory Bulb. Brain Struct. Funct..

[83] Danielson N., Byrne M. (2020). Indications for Allogeneic Hematopoietic Cell Transplantation in Myelodysplastic Syndrome. Curr. Hematol. Malig. Rep..

[84] Mohammadi R., Aryan A., Omrani M.D., Ghaderian S.M.H., Fazeli Z. (2021). Autologous Hematopoietic Stem Cell Transplantation (AHSCT): An Evolving Treatment Avenue in Multiple Sclerosis. Biologics.

[85] Oliveira M.C., Elias J.B., de Moraes D.A., Simões B.P., Rodrigues M., Ribeiro A.A.F., Piron-Ruiz L., Ruiz M.A., Hamerschlak N. (2021). A Review of Hematopoietic Stem Cell Transplantation for Autoimmune Diseases: Multiple Sclerosis, Systemic Sclerosis and Crohn’s Disease. Position Paper of the Brazilian Society of Bone Marrow Transplantation. Hematol. Transfus. Cell Ther..

[86] Nikoonezhad M., Lasemi M.V., Alamdari S., Mohammadian M., Tabarraee M., Ghadyani M., Hamidpour M., Roshandel E. (2022). Treatment of Insulin-Dependent Diabetes by Hematopoietic Stem Cell Transplantation. Transpl. Immunol..

[87] Koda M., Okada S., Nakayama T., Koshizuka S., Kamada T., Nishio Y., Someya Y., Yoshinaga K., Okawa A., Moriya H. (2005). Hematopoietic Stem Cell and Marrow Stromal Cell for Spinal Cord Injury in Mice. Neuroreport.

[88] Ozdemir Z.N., Civriz Bozdağ S. (2018). Graft Failure after Allogeneic Hematopoietic Stem Cell Transplantation. Transfus. Apher. Sci..

[89] Deda H., Inci M.C., Kurekçi A., Kayihan K., Özgün E., Ustunsoy G., Kocabay S. (2008). Treatment of Chronic Spinal Cord Injured Patients with Autologous Bone Marrow-Derived Hematopoietic Stem Cell Transplantation: 1-Year Follow-Up. Cytotherapy.

[90] Müller A.M., Huppertz S., Henschler R. (2016). Hematopoietic Stem Cells in Regenerative Medicine: Astray or on the Path?. Transfus. Med. Hemotherapy.

[91] Mosaad Y.M. (2014). Hematopoietic Stem Cells: An Overview. Transfus. Apher. Sci..

[92] Hou Y., Liu X., Guo Y., Liu D., Guo P., Liu J. (2022). Strategies for Effective Neural Circuit Reconstruction After Spinal Cord Injury: Use of Stem Cells and Biomaterials. World Neurosurg..

[93] Zhao A., Chung M., Yang Y., Pan X., Pan Y., Cai S. (2022). The SDF-1/CXCR4 Signaling Pathway Directs the Migration of Systemically Transplanted Bone Marrow Mesenchymal Stem Cells towards the Lesion Site in a Rat Model of Spinal Cord Injury. Curr. Stem Cell Res. Ther..

[94] Pelagalli A., Nardelli A., Lucarelli E., Zannetti A., Brunetti A. (2018). Autocrine Signals Increase Ovine Mesenchymal Stem Cells Migration through Aquaporin-1 and CXCR4 Overexpression. J. Cell. Physiol..

[95] Marquez-Curtis L.A., Gul-Uludag H., Xu P., Chen J., Janowska-Wieczorek A. (2013). CXCR4 Transfection of Cord Blood Mesenchymal Stromal Cells with the Use of Cationic Liposome Enhances Their Migration toward Stromal Cell-Derived Factor-1. Cytotherapy.

[96] Xie J.-L., Wang X.-R., Li M.-M., Tao Z.-H., Teng W.-W. (2022). Saijilafu Mesenchymal Stromal Cell Therapy in Spinal Cord Injury: Mechanisms and Prospects. Front. Cell. Neurosci..

[97] Bang O.Y., Moon G.J., Kim D.H., Lee J.H., Kim S., Son J.P., Cho Y.H., Chang W.H., Kim Y.H., Sung J.H. (2017). Stroke Induces Mesenchymal Stem Cell Migration to Infarcted Brain Areas Via CXCR4 and C-Met Signaling. Transl. Stroke Res..

[98] He W., Shi C., Yin J., Huang F., Yan W., Deng J., Zhang B., Wang B., Wang H. (2022). Spinal Cord Decellularized Matrix Scaffold Loaded with Engineered Basic Fibroblast Growth Factor-Overexpressed Human Umbilical Cord Mesenchymal Stromal Cells Promoted the Recovery of Spinal Cord Injury. J. Biomed. Mater. Res. Part B Appl. Biomater..

[99] Song P., Han T., Xiang X., Wang Y., Fang H., Niu Y., Shen C. (2020). The Role of Hepatocyte Growth Factor in Mesenchymal Stem Cell-Induced Recovery in Spinal Cord Injured Rats. Stem Cell Res. Ther..

[100] Zhang Y., Yang J., Zhang P., Liu T., Xu J., Fan Z., Shen Y., Li W., Zhang H. (2016). Calcitonin Gene-Related Peptide Is a Key Factor in the Homing of Transplanted Human MSCs to Sites of Spinal Cord Injury. Sci. Rep..

[101] Fu X., Liu G., Halim A., Ju Y., Luo Q., Song G. (2019). Mesenchymal Stem Cell Migration and Tissue Repair. Cells.

[102] Nitzsche F., Müller C., Lukomska B., Jolkkonen J., Deten A., Boltze J. (2017). Concise Review: MSC Adhesion Cascade-Insights into Homing and Transendothelial Migration. Stem Cells.

[103] Nam D., Park A., Dubon M.J., Yu J., Kim W., Son Y., Park K.S. (2020). Coordinated Regulation of Mesenchymal Stem Cell Migration by Various Chemotactic Stimuli. Int. J. Mol. Sci..

[104] Rahimi-Sherbaf F., Nadri S., Nadri S., Rahmani A., Oskoei A.D. (2020). Placenta Mesenchymal Stem Cells Differentiation toward Neuronal-like Cells on Nanofibrous Scaffold. BioImpacts.

[105] Zhang K., Liu Z., Li G., Lai B.Q., Qin L.N., Ding Y., Ruan J.W., Zhang S.X., Zeng Y.S. (2014). Electro-Acupuncture Promotes the Survival and Differentiation of Transplanted Bone Marrow Mesenchymal Stem Cells Pre-Induced with Neurotrophin-3 and Retinoic Acid in Gelatin Sponge Scaffold after Rat Spinal Cord Transection. Stem Cell Rev. Rep..

[106] Wang C., Shi D., Song X., Chen Y., Wang L., Zhang X. (2016). Calpain Inhibitor Attenuates ER Stress-Induced Apoptosis in Injured Spinal Cord after Bone Mesenchymal Stem Cells Transplantation. Neurochem. Int..

[107] Chung H.J., Chung W.H., Lee J.H., Chung D.J., Yang W.J., Lee A.J., Choi C.B., Chang H.S., Kim D.H., Suh H.J. (2016). Expression of Neurotrophic Factors in Injured Spinal Cord after Transplantation of Human-Umbilical Cord Blood Stem Cells in Rats. J. Vet. Sci..

[108] Kim Y., Jo S.H., Kim W.H., Kweon O.K. (2015). Antioxidant and Anti-Inflammatory Effects of Intravenously Injected Adipose Derived Mesenchymal Stem Cells in Dogs with Acute Spinal Cord Injury. Stem Cell Res. Ther..

[109] Phinney D.G., Pittenger M.F. (2017). Concise Review: MSC-Derived Exosomes for Cell-Free Therapy. Stem Cells.

[110] Pittenger M.F., Discher D.E., Péault B.M., Phinney D.G., Hare J.M., Caplan A.I. (2019). Mesenchymal Stem Cell Perspective: Cell Biology to Clinical Progress. NPJ Regen. Med..

[111] Tahmasebi F., Barati S. (2022). Effects of Mesenchymal Stem Cell Transplantation on Spinal Cord Injury Patients. Cell Tissue Res..

[112] Martins L.F., Costa R.O., Pedro J.R., Aguiar P., Serra S.C., Teixeira F.G., Sousa N., Salgado A.J., Almeida R.D. (2017). Mesenchymal Stem Cells Secretome-Induced Axonal Outgrowth Is Mediated by BDNF. Sci. Rep..

[113] Chang D.J., Cho H.Y., Hwang S., Lee N., Choi C., Lee H., Hong K.S., Oh S.H., Kim H.S., Shin D.A. (2021). Therapeutic Effect of BDNF-Overexpressing Human Neural Stem Cells (F3.BDNF) in a Contusion Model of Spinal Cord Injury in Rats. Int. J. Mol. Sci..

[114] Sieck G.C., Gransee H.M., Zhan W.Z., Mantilla C.B. (2021). Neural Circuits: Acute Intrathecal BDNF Enhances Functional Recovery after Cervical Spinal Cord Injury in Rats. J. Neurophysiol..

[115] Walker M.J., Xu X.M. (2018). History of Glial Cell Line-Derived Neurotrophic Factor (GDNF) and Its Use for Spinal Cord Injury Repair. Brain Sci..

[116] Pajer K., Bellák T., Nógrádi A. (2021). Stem Cell Secretome for Spinal Cord Repair: Is It More than Just a Random Baseline Set of Factors?. Cells.

[117] Sivak W.N., White J.D., Bliley J.M., Tien L.W., Liao H.T., Kaplan D.L., Marra K.G. (2017). Delivery of Chondroitinase ABC and Glial Cell Line-Derived Neurotrophic Factor from Silk Fibroin Conduits Enhances Peripheral Nerve Regeneration. J. Tissue Eng. Regen. Med..

[118] Kitamura K., Nagoshi N., Tsuji O., Matsumoto M., Okano H., Nakamura M. (2019). Application of Hepatocyte Growth Factor for Acute Spinal Cord Injury: The Road from Basic Studies to Human Treatment. Int. J. Mol. Sci..

[119] Agrelo I.S., Schira-Heinen J., Beyer F., Groh J., Bütermann C., Estrada V., Poschmann G., Bribian A., Jadasz J.J., Lopez-Mascaraque L. (2020). Secretome Analysis of Mesenchymal Stem Cell Factors Fostering Oligodendroglial Differentiation of Neural Stem Cells In Vivo. Int. J. Mol. Sci..

[120] Zhang Y., Yang S., Liu C., Han X., Gu X., Zhou S. (2021). Deciphering Glial Scar after Spinal Cord Injury. Burn. Trauma.

[121] Kim M., Kim K.H., Song S.U., Yi T.G., Yoon S.H., Park S.R., Choi B.H. (2018). Transplantation of Human Bone Marrow-Derived Clonal Mesenchymal Stem Cells Reduces Fibrotic Scar Formation in a Rat Spinal Cord Injury Model. J. Tissue Eng. Regen. Med..

[122] Huang F., Gao T., Wang W., Wang L., Xie Y., Tai C., Liu S., Cui Y., Wang B. (2021). Engineered Basic Fibroblast Growth Factor-Overexpressing Human Umbilical Cord-Derived Mesenchymal Stem Cells Improve the Proliferation and Neuronal Differentiation of Endogenous Neural Stem Cells and Functional Recovery of Spinal Cord Injury by Activating the PI3K-Akt-GSK-3β Signaling Pathway. Stem Cell Res. Ther..

[123] Pang Q.M., Chen S.Y., Xu Q.J., Fu S.P., Yang Y.C., Zou W.H., Zhang M., Liu J., Wan W.H., Peng J.C. (2021). Neuroinflammation and Scarring After Spinal Cord Injury: Therapeutic Roles of MSCs on Inflammation and Glial Scar. Front. Immunol..

[124] Lv C., Zhang T., Li K., Gao K. (2020). Bone Marrow Mesenchymal Stem Cells Improve Spinal Function of Spinal Cord Injury in Rats via TGF-Beta/Smads Signaling Pathway. Exp. Ther. Med..

[125] Kim C., Kim H.J., Lee H., Lee H., Lee S.J., Lee S.T., Yang S.-R., Chung C.K. (2019). Mesenchymal Stem Cell Transplantation Promotes Functional Recovery through MMP2/STAT3 Related Astrogliosis after Spinal Cord Injury. Int. J. Stem Cells.

[126] Yang Y., Cao T.T., Tian Z.M., Gao H., Wen H.Q., Pang M., He W.J., Wang N.X., Chen Y.Y., Wang Y. (2020). Subarachnoid Transplantation of Human Umbilical Cord Mesenchymal Stem Cell in Rodent Model with Subacute Incomplete Spinal Cord Injury: Preclinical Safety and Efficacy Study. Exp. Cell Res..

[127] Fu Q., Liu Y., Liu X., Zhang Q., Chen L., Peng J., Ao J., Li Y., Wang S., Song G. (2017). Engrafted Peripheral Blood-Derived Mesenchymal Stem Cells Promote Locomotive Recovery in Adult Rats after Spinal Cord Injury. Am. J. Transl. Res..

[128] Cao Y., Xu Y., Chen C., Xie H., Lu H., Hu J. (2021). Local Delivery of USC-Derived Exosomes Harboring ANGPTL3 Enhances Spinal Cord Functional Recovery after Injury by Promoting Angiogenesis. Stem Cell Res. Ther..

[129] Zhong D., Cao Y., Li C.J., Li M., Rong Z.J., Jiang L., Guo Z., Lu H.B., Hu J.Z. (2020). Highlight Article: Neural Stem Cell-Derived Exosomes Facilitate Cord Functional Recovery after Injury by Promoting. Exp. Biol. Med..

[130] Al Mamun A., Monalisa I., Tul Kubra K., Akter A., Akter J., Sarker T., Munir F., Wu Y., Jia C., Afrin Taniya M. (2021). Advances in Immunotherapy for the Treatment of Spinal Cord Injury. Immunobiology.

[131] Urdzíková L.M., Růžička J., LaBagnara M., Kárová K., Kubinová Š., Jiráková K., Murali R., Syková E., Jhanwar-Uniyal M., Jendelová P. (2014). Human Mesenchymal Stem Cells Modulate Inflammatory Cytokines after Spinal Cord Injury in Rat. Int. J. Mol. Sci..

[132] Hakim R., Covacu R., Zachariadis V., Frostell A., Sankavaram S.R., Brundin L., Svensson M. (2019). Mesenchymal Stem Cells Transplanted into Spinal Cord Injury Adopt Immune Cell-like Characteristics. Stem Cell Res. Ther..

[133] Glennie S., Soeiro I., Dyson P.J., Lam E.W.F., Dazzi F. (2005). Bone Marrow Mesenchymal Stem Cells Induce Division Arrest Anergy of Activated T Cells. Blood.

[134] Volarevic V., Gazdic M., Simovic Markovic B., Jovicic N., Djonov V., Arsenijevic N. (2017). Mesenchymal Stem Cell-Derived Factors: Immuno-Modulatory Effects and Therapeutic Potential. Biofactors.

[135] Wang Q., Yang Q., Wang Z., Tong H., Ma L., Zhang Y., Shan F., Meng Y., Yuan Z. (2016). Comparative Analysis of Human Mesenchymal Stem Cells from Fetal-Bone Marrow, Adipose Tissue, and Warton’s Jelly as Sources of Cell Immunomodulatory Therapy. Hum. Vaccin. Immunother..

[136] Wang L., Pei S., Han L., Guo B., Li Y., Duan R., Yao Y., Xue B., Chen X., Jia Y. (2018). Mesenchymal Stem Cell-Derived Exosomes Reduce A1 Astrocytes via Downregulation of Phosphorylated NFκB P65 Subunit in Spinal Cord Injury. Cell. Physiol. Biochem. Int. J. Exp. Cell. Physiol. Biochem. Pharmacol..

[137] An N., Yang J., Wang H., Sun S., Wu H., Li L., Li M. (2021). Mechanism of Mesenchymal Stem Cells in Spinal Cord Injury Repair through Macrophage Polarization. Cell Biosci..

[138] Wu L.-L., Pan X.-M., Chen H.-H., Fu X.-Y., Jiang J., Ding M.-X. (2020). Repairing and Analgesic Effects of Umbilical Cord Mesenchymal Stem Cell Transplantation in Mice with Spinal Cord Injury. Biomed Res. Int..

[139] Wang B., Chang M., Zhang R., Wo J., Wu B., Zhang H., Zhou Z., Li Z., Zhang F., Zhong C. (2022). Spinal Cord Injury Target-Immunotherapy with TNF-α Autoregulated and Feedback-Controlled Human Umbilical Cord Mesenchymal Stem Cell Derived Exosomes Remodelled by CRISPR/Cas9 Plasmid. Biomater. Adv..

[140] Litak J., Szymoniuk M., Czyżewski W., Hoffman Z., Litak J., Sakwa L., Kamieniak P. (2022). Metallic Implants Used in Lumbar Interbody Fusion. Materials.

[141] Litak J., Czyzewski W., Szymoniuk M., Pastuszak B., Litak J., Litak G., Grochowski C., Rahnama-Hezavah M., Kamieniak P. (2022). Hydroxyapatite Use in Spine Surgery&mdash;Molecular and Clinical Aspect. Materials.

[142] Chung H., Park S. (2016). Ghrelin Regulates Cell Cycle-Related Gene Expression in Cultured Hippocampal Neural Stem Cells. J. Endocrinol..

[143] Glass J.D., Hertzberg V.S., Boulis N.M., Riley J., Federici T., Polak M., Bordeau J., Fournier C., Johe K., Hazel T. (2016). Transplantation of Spinal Cord–Derived Neural Stem Cells for ALS. Neurology.

[144] Todd K.L., Baker K.L., Eastman M.B., Kolling F.W., Trausch A.G., Nelson C.E., Conover J.C. (2017). EphA4 Regulates Neuroblast and Astrocyte Organization in a Neurogenic Niche. J. Neurosci..

[145] Mardones M.D., Andaur G.A., Varas-Godoy M., Henriquez J.F., Salech F., Behrens M.I., Couve A., Inestrosa N.C., Varela-Nallar L. (2016). Frizzled-1 Receptor Regulates Adult Hippocampal Neurogenesis. Mol. Brain.

[146] Meneghini V., Frati G., Sala D., De Cicco S., Luciani M., Cavazzin C., Paulis M., Mentzen W., Morena F., Giannelli S. (2017). Generation of Human Induced Pluripotent Stem Cell-Derived Bona Fide Neural Stem Cells for Ex Vivo Gene Therapy of Metachromatic Leukodystrophy. Stem Cells Transl. Med..

[147] Morell M., Tsan Y., O’Shea K.S. (2015). Inducible Expression of Noggin Selectively Expands Neural Progenitors in the Adult SVZ. Stem Cell Res..

[148] Wang J., Fu X., Zhang D., Yu L., Li N., Lu Z., Gao Y., Wang M., Liu X., Zhou C. (2017). ChAT-Positive Neurons Participate in Subventricular Zone Neurogenesis after Middle Cerebral Artery Occlusion in Mice. Behav. Brain Res..

[149] Yu X., Li Z., Zheng H., Ho J., Chan M.T.V., Wu W.K.K. (2017). Protective Roles of Melatonin in Central Nervous System Diseases by Regulation of Neural Stem Cells. Cell Prolif..

[150] Zhang M., Lin Y.H., Sun Y.J., Zhu S., Zheng J., Liu K., Cao N., Li K., Huang Y., Ding S. (2016). Pharmacological Reprogramming of Fibroblasts into Neural Stem Cells by Signaling-Directed Transcriptional Activation. Cell Stem Cell.

[151] Aggarwal R., Lu J., Pompili V.J., Das H. (2012). Hematopoietic Stem Cells: Transcriptional Regulation, Ex Vivo Expansion and Clinical Application. Curr. Mol. Med..

[152] Frolov A.A., Bryukhovetskiy A.S. (2012). Effects of Hematopoietic Autologous Stem Cell Transplantation to the Chronically Injured Human Spinal Cord Evaluated by Motor and Somatosensory Evoked Potentials Methods. Cell Transplant..

[153] Moghaddam S.A., Yousefi B., Sanooghi D., Faghihi F., Hayati Roodbari N., Bana N., Joghataei M.T., Pooyan P., Arjmand B. (2017). Differentiation Potential of Human CD133 Positive Hematopoietic Stem Cells into Motor Neuron- like Cells, in Vitro. J. Chem. Neuroanat..

[154] Xiong L.L., Liu F., Deng S.K., Liu J., Dan Q.Q., Zhang P., Zou Y., Xia Q.J., Wang T.H. (2017). Transplantation of Hematopoietic Stem Cells Promotes Functional Improvement Associated with NT-3-MEK-1 Activation in Spinal Cord-Transected Rats. Front. Cell. Neurosci..

[155] Takakura N., Watanabe T., Suenobu S., Yamada Y., Noda T., Ito Y., Satake M., Suda T. (2000). A Role for Hematopoietic Stem Cells in Promoting Angiogenesis. Cell.

[156] Liu Y., Kelamangalath L., Kim H., Han S.B., Tang X., Zhai J., Hong J.W., Lin S., Son Y.J., Smith G.M. (2016). NT-3 Promotes Proprioceptive Axon Regeneration When Combined with Activation of the MTor Intrinsic Growth Pathway but Not with Reduction of Myelin Extrinsic Inhibitors. Exp. Neurol..

[157] Keefe K.M., Sheikh I.S., Smith G.M. (2017). Targeting Neurotrophins to Specific Populations of Neurons: NGF, BDNF, and NT-3 and Their Relevance for Treatment of Spinal Cord Injury. Int. J. Mol. Sci..

[158] Ataei M.L., Karimipour M., Shahabi P., Pashaei-Asl R., Ebrahimie E., Pashaiasl M. (2021). The Restorative Effect of Human Amniotic Fluid Stem Cells on Spinal Cord Injury. Cells.

[159] Satti H.S., Waheed A., Ahmed P., Ahmed K., Akram Z., Aziz T., Satti T.M., Shahbaz N., Khan M.A., Malik S.A. (2016). Autologous Mesenchymal Stromal Cell Transplantation for Spinal Cord Injury: A Phase I Pilot Study. Cytotherapy.

[160] Vaquero J., Zurita M., Rico M.A., Aguayo C., Fernández C., Gutiérrez R., Rodríguez-Boto G., Saab A., Hassan R., Ortega C. (2018). Intrathecal Administration of Autologous Bone Marrow Stromal Cells Improves Neuropathic Pain in Patients with Spinal Cord Injury. Neurosci. Lett..

[161] Chen X., Xue B., Li Y., Song C., Jia P., Ren X., Zang W., Wang J. (2017). Meta-Analysis of Stem Cell Transplantation for Reflex Hypersensitivity after Spinal Cord Injury. Neuroscience.

[162] Kazim S.F., Bowers C.A., Cole C.D., Varela S., Karimov Z., Martinez E., Ogulnick J.V., Schmidt M.H. (2021). Corticospinal Motor Circuit Plasticity After Spinal Cord Injury: Harnessing Neuroplasticity to Improve Functional Outcomes. Mol. Neurobiol..

[163] Zamani H., Soufizomorrod M., Oraee-Yazdani S., Naviafar D., Akhlaghpasand M., Seddighi A., Soleimani M. (2022). Safety and Feasibility of Autologous Olfactory Ensheathing Cell and Bone Marrow Mesenchymal Stem Cell Co-Transplantation in Chronic Human Spinal Cord Injury: A Clinical Trial. Spinal Cord.

[164] Oraee-Yazdani S., Akhlaghpasand M., Golmohammadi M., Hafizi M., Zomorrod M.S., Kabir N.M., Oraee-Yazdani M., Ashrafi F., Zali A., Soleimani M. (2021). Combining Cell Therapy with Human Autologous Schwann Cell and Bone Marrow-Derived Mesenchymal Stem Cell in Patients with Subacute Complete Spinal Cord Injury: Safety Considerations and Possible Outcomes. Stem Cell Res. Ther..

[165] Deng W.S., Ma K., Liang B., Liu X.Y., Xu H.Y., Zhang J., Shi H.Y., Sun H.T., Chen X.Y., Zhang S. (2020). Collagen Scaffold Combined with Human Umbilical Cord-Mesenchymal Stem Cells Transplantation for Acute Complete Spinal Cord Injury. Neural Regen. Res..

[166] Sharma A., Sane H., Gokulchandran N., Kulkarni P., Jose A., Nair V., Das R., Lakhanpal V., Badhe P. (2020). Intrathecal Transplantation of Autologous Bone Marrow Mononuclear Cells in Patients with Sub-Acute and Chronic Spinal Cord Injury: An Open-Label Study. Int. J. Health Sci..

[167] Levi A.D., Okonkwo D.O., Park P., Jenkins A.L., Kurpad S.N., Parr A.M., Ganju A., Aarabi B., Kim D., Casha S. (2018). Emerging Safety of Intramedullary Transplantation of Human Neural Stem Cells in Chronic Cervical and Thoracic Spinal Cord Injury. Neurosurgery.

[168] Curtis E., Martin J.R., Gabel B., Sidhu N., Rzesiewicz T.K., Mandeville R., Van Gorp S., Leerink M., Tadokoro T., Marsala S. (2018). A First-in-Human, Phase I Study of Neural Stem Cell Transplantation for Chronic Spinal Cord Injury. Cell Stem Cell.

[169] Xiao Z., Tang F., Zhao Y., Han G., Yin N., Li X., Chen B., Han S., Jiang X., Yun C. (2018). Significant Improvement of Acute Complete Spinal Cord Injury Patients Diagnosed by a Combined Criteria Implanted with NeuroRegen Scaffolds and Mesenchymal Stem Cells. Cell Transplant..

[170] Vaquero J., Zurita M., Rico M.A., Bonilla C., Aguayo C., Fernández C., Tapiador N., Sevilla M., Morejón C., Montilla J. (2017). Repeated Subarachnoid Administrations of Autologous Mesenchymal Stromal Cells Supported in Autologous Plasma Improve Quality of Life in Patients Suffering Incomplete Spinal Cord Injury. Cytotherapy.

[171] Ammar A.S., Osman Y., Hendam A.T., Hasen M.A., Al Rubaish F.A., Al Nujaidi D.Y., Al Abbas F.M. (2017). A Method for Reconstruction of Severely Damaged Spinal Cord Using Autologous Hematopoietic Stem Cells and Platelet-Rich Protein as a Biological Scaffold. Asian J. Neurosurg..

[172] Liu S., Zhang H., Wang H., Huang J., Yang Y., Li G., Yu K., Yang L. (2022). A Comparative Study of Different Stem Cell Transplantation for Spinal Cord Injury: A Systematic Review and Network Meta-Analysis. World Neurosurg..

[173] Chen W., Liu W., Bai Y., Zhou Y., Zhang Y., Wang C., Lin S., He H. (2021). Transplantation of Mesenchymal Stem Cells for Spinal Cord Injury: A Systematic Review and Network Meta-Analysis. J. Transl. Med..

[174] Tang Q.R., Xue H., Zhang Q., Guo Y., Xu H., Liu Y., Liu J.M. (2021). Evaluation of the Clinical Efficacy of Stem Cell Transplantation in the Treatment of Spinal Cord Injury: A Systematic Review and Meta-Analysis. Cell Transplant..

[175] Xu P., Yang X. (2019). The Efficacy and Safety of Mesenchymal Stem Cell Transplantation for Spinal Cord Injury Patients: A Meta-Analysis and Systematic Review. Cell Transplant..

[176] Fan X., Wang J.Z., Lin X.M., Zhang L. (2017). Stem Cell Transplantation for Spinal Cord Injury: A Meta-Analysis of Treatment Effectiveness and Safety. Neural Regen. Res..

[177] Maqueda A., Rodriguez F.J. (2020). Efficacy of Human HC016 Cell Transplants on Neuroprotection and Functional Recovery in a Rat Model of Acute Spinal Cord Injury. J. Tissue Eng. Regen. Med..

[178] Dasari V.R., Spomar D.G., Li L., Gujrati M., Rao J.S., Dinh D.H. (2008). Umbilical Cord Blood Stem Cell Mediated Downregulation of Fas Improves Functional Recovery of Rats after Spinal Cord Injury. Neurochem. Res..

[179] Cabanes C., Bonilla S., Tabares L., Martínez S. (2007). Neuroprotective Effect of Adult Hematopoietic Stem Cells in a Mouse Model of Motoneuron Degeneration. Neurobiol. Dis..

[180] Bryukhovetskiy A.S. (2015). Effectiveness of Repeated Transplantations of Hematopoietic Stem Cells in Spinal Cord Injury. World J. Transplant..

[181] Thakkar U., Vanikar A., Trivedi H., Shah V., Dave S., Dixit S., Tiwari B., Shah H. (2016). Infusion of Autologous Adipose Tissue Derived Neuronal Differentiated Mesenchymal Stem Cells and Hematopoietic Stem Cells in Post-Traumatic Paraplegia Offers a Viable Therapeutic Approach. Adv. Biomed. Res..

[182] Zakerinia M., Kamgarpour A., Nemati H., Zare H.R., Ghasemfar M., Rezvani A.R., Karimi M., Nourani Khojasteh H., Dehghani M., Vojdani R. (2018). Intrathecal Autologous Bone Marrow-Derived Hematopoietic Stem Cell Therapy in Neurological Diseases. Int. J. Organ Transplant. Med..

[183] Dryla A., Szymoniuk M., Rogatko K., Piecewicz-Szczęsna H. (2022). Alveolar Osteitis: The Current State of Knowledge. J. Educ. Health Sport.

[184] Sharara F.I., Lelea L.L., Rahman S., Klebanoff J.S., Moawad G.N. (2021). A Narrative Review of Platelet-Rich Plasma (PRP) in Reproductive Medicine. J. Assist. Reprod. Genet..

[185] Hajipour H., Farzadi L., Latifi Z., Keyhanvar N., Navali N., Fattahi A., Nouri M., Dittrich R. (2021). An Update on Platelet-Rich Plasma (PRP) Therapy in Endometrium and Ovary Related Infertilities: Clinical and Molecular Aspects. Syst. Biol. Reprod. Med..

[186] Garcia-Ayuso D., Di Pierdomenico J., García-Bernal D., Vidal-Sanz M., Villegas-Pérez M.P. (2022). Bone Marrow-Derived Mononuclear Stem Cells in the Treatment of Retinal Degenerations. Neural Regen. Res..

[187] dos Ramalho B.S., de Almeida F.M., Martinez A.M.B. (2021). Cell Therapy and Delivery Strategies for Spinal Cord Injury. Histol. Histopathol..

[188] Veneruso V., Rossi F., Villella A., Bena A., Forloni G., Veglianese P. (2019). Stem Cell Paracrine Effect and Delivery Strategies for Spinal Cord Injury Regeneration. J. Control. Release.

[189] Mukhamedshina Y.O., Gracheva O.A., Mukhutdinova D.M., Chelyshev Y.A., Rizvanov A.A. (2019). Mesenchymal Stem Cells and the Neuronal Microenvironment in the Area of Spinal Cord Injury. Neural Regen. Res..

[190] Zhu H., Poon W., Liu Y., Leung G.K.-K., Wong Y., Feng Y., Ng S.C.P., Tsang K.S., Sun D.T.F., Yeung D.K. (2016). Phase I-II Clinical Trial Assessing Safety and Efficacy of Umbilical Cord Blood Mononuclear Cell Transplant Therapy of Chronic Complete Spinal Cord Injury. CELL Transplant..

[191] Krupa P., Vackova I., Ruzicka J., Zaviskova K., Dubisova J., Koci Z., Turnovcova K., Urdzikova L.M., Kubinova S., Rehak S. (2018). The Effect of Human Mesenchymal Stem Cells Derived from Wharton’s Jelly in Spinal Cord Injury Treatment Is Dose-Dependent and Can Be Facilitated by Repeated Application. Int. J. Mol. Sci..

[192] Bansal H., Verma P., Agrawal A., Leon J., Sundell I.B., Koka P.S. (2016). Autologous Bone Marrow-Derived Stem Cells in Spinal Cord Injury. J. Stem Cells.

[193] Li J., Chen L., Chen Q., Hu D., Lin J. (2019). Effect of Granulocyte Colony-Stimulating Factor Mobilizing Bone Marrow Mesenchymal Stell Cells Homing to Injury Sites in Spinal Cord Injury of Rats. Zhongguo Xiu Fu Chong Jian Wai Ke Za Zhi.

[194] Kabat M., Bobkov I., Kumar S., Grumet M. (2020). Trends in Mesenchymal Stem Cell Clinical Trials 2004-2018: Is Efficacy Optimal in a Narrow Dose Range?. Stem Cells Transl. Med..

[195] Ferrini E., Stellari F.F., Franceschi V., Macchi F., Russo L., Murgia A., Grisendi G., Villetti G., Dominici M., Donofrio G. (2021). Persistency of Mesenchymal Stromal/Stem Cells in Lungs. Front. Cell Dev. Biol..

[196] Moll G., Ankrum J.A., Kamhieh-Milz J., Bieback K., Ringdén O., Volk H.D., Geissler S., Reinke P. (2019). Intravascular Mesenchymal Stromal/Stem Cell Therapy Product Diversification: Time for New Clinical Guidelines. Trends Mol. Med..

[197] Ramalho B.D.S., De Almeida F.M., Sales C.M., De Lima S., Martinez A.M.B. (2018). Injection of Bone Marrow Mesenchymal Stem Cells by Intravenous or Intraperitoneal Routes Is a Viable Alternative to Spinal Cord Injury Treatment in Mice. Neural Regen. Res..

[198] Oh S.K., Jeon S.R. (2016). Current Concept of Stem Cell Therapy for Spinal Cord Injury: A Review. Korean J. Neurotrauma.

[199] Boido M., Ghibaudi M., Gentile P., Favaro E., Fusaro R., Tonda-Turo C. (2019). Chitosan-Based Hydrogel to Support the Paracrine Activity of Mesenchymal Stem Cells in Spinal Cord Injury Treatment. Sci. Rep..

[200] Liu W., Wang Y., Gong F., Rong Y., Luo Y., Tang P., Zhou Z., Zhou Z., Xu T., Jiang T. (2019). Exosomes Derived from Bone Mesenchymal Stem Cells Repair Traumatic Spinal Cord Injury by Suppressing the Activation of A1 Neurotoxic Reactive Astrocytes. J. Neurotrauma.

[201] Okuda A., Horii-Hayashi N., Sasagawa T., Shimizu T., Shigematsu H., Iwata E., Morimoto Y., Masuda K., Koizumi M., Akahane M. (2017). Bone Marrow Stromal Cell Sheets May Promote Axonal Regeneration and Functional Recovery with Suppression of Glial Scar Formation after Spinal Cord Transection Injury in Rats. J. Neurosurg. Spine.

[202] Cheng I., Park D.Y., Mayle R.E., Githens M., Smith R.L., Park H.Y., Hu S.S., Alamin T.F., Wood K.B., Kharazi A.I. (2017). Does Timing of Transplantation of Neural Stem Cells Following Spinal Cord Injury Affect Outcomes in an Animal Model?. J. Spine Surg..

[203] Shang Z., Li D., Chen J., Wang R.R., Wang M., Zhang B., Wang X., Wanyan P. (2022). What Is the Optimal Timing of Transplantation of Neural Stem Cells in Spinal Cord Injury? A Systematic Review and Network Meta-Analysis Based on Animal Studies. Front. Immunol..

[204] de Araújo L.T., Macêdo C.T., Damasceno P.K.F., Das Neves Í.G.C., de Lima C.S., Santos G.C., de Santana T.A., Sampaio G.L.d.A., Silva D.N., Villarreal C.F. (2022). Clinical Trials Using Mesenchymal Stem Cells for Spinal Cord Injury: Challenges in Generating Evidence. Cells.

[205] Vaquero J., Zurita M., Rico M.A., Bonilla C., Aguayo C., Montilla J., Bustamante S., Carballido J., Marin E., Martinez F. (2016). An Approach to Personalized Cell Therapy in Chronic Complete Paraplegia: The Puerta de Hierro Phase I/II Clinical Trial. Cytotherapy.

[206] Liu W., Ma Z., Li J., Kang X. (2021). Mesenchymal Stem Cell-Derived Exosomes: Therapeutic Opportunities and Challenges for Spinal Cord Injury. Stem Cell Res. Ther..

[207] Liang Y., Wu J.-H., Zhu J.-H., Yang H. (2022). Exosomes Secreted by Hypoxia-Pre-Conditioned Adipose-Derived Mesenchymal Stem Cells Reduce Neuronal Apoptosis in Rats with Spinal Cord Injury. J. Neurotrauma.

[208] Koprivec S., Novak M., Bernik S., Voga M., Mohorič L., Majdič G. (2021). Treatment of Cranial Cruciate Ligament Injuries in Dogs Using a Combination of Tibial Tuberosity Advancement Procedure and Autologous Mesenchymal Stem Cells/Multipotent Mesenchymal Stromal Cells—A Pilot Study. Acta Vet. Hung..

[209] Chen Y., Tian Z., He L., Liu C., Wang N., Rong L., Liu B. (2021). Exosomes Derived from MiR-26a-Modified MSCs Promote Axonal Regeneration via the PTEN/AKT/MTOR Pathway Following Spinal Cord Injury. Stem Cell Res. Ther..

[210] Herbert F.J., Bharathi D., Suresh S., David E., Kumar S. (2022). Regenerative Potential of Stem Cell-Derived Extracellular Vesicles in Spinal Cord Injury (SCI). Curr. Stem Cell Res. Ther..

[211] Yousefifard M., Sarveazad A., Babahajian A., Rafiei Alavi S.N., Madani Neishaboori A., Vaccaro A.R., Hosseini M., Rahimi-Movaghar V. (2022). Growth Factor Gene-Modified Cells in Spinal Cord Injury Recovery: A Systematic Review. World Neurosurg..

[212] Lu D., Yang Y., Zhang P., Ma Z., Li W., Song Y., Feng H., Yu W., Ren F., Li T. (2022). Development and Application of Three-Dimensional Bioprinting Scaffold in the Repair of Spinal Cord Injury. Tissue Eng. Regen. Med..

[213] Zhou Y., Wen L.L., Li Y.F., Wu K.M., Duan R.R., Yao Y.B., Jing L.J., Gong Z., Teng J.F., Jia Y.J. (2022). Exosomes Derived from Bone Marrow Mesenchymal Stem Cells Protect the Injured Spinal Cord by Inhibiting Pericyte Pyroptosis. Neural Regen. Res..

[214] Tian F., Yang J., Xia R. (2022). Exosomes Secreted from CircZFHX3-Modified Mesenchymal Stem Cells Repaired Spinal Cord Injury Through Mir-16-5p/IGF-1 in Mice. Neurochem. Res..

[215] Shao C., Chen Y., Yang T., Zhao H., Li D. (2022). Mesenchymal Stem Cell Derived Exosomes Suppress Neuronal Cell Ferroptosis Via IncGm36569/MiR-5627-5p/FSP1 Axis in Acute Spinal Cord Injury. Stem Cell Rev. Rep..

[216] Kang J., Guo Y. (2022). Human Umbilical Cord Mesenchymal Stem Cells Derived Exosomes Promote Neurological Function Recovery in a Rat Spinal Cord Injury Model. Neurochem. Res..

[217] Zhao Y., Chen Y., Wang Z., Xu C., Qiao S., Liu T., Qi K., Tong D., Li C. (2022). Bone Marrow Mesenchymal Stem Cell Exosome Attenuates Inflammasome-Related Pyroptosis via Delivering Circ_003564 to Improve the Recovery of Spinal Cord Injury. Mol. Neurobiol..

[218] Zhang C., Deng R., Zhang G., He X., Chen H., Chen B., Wan L., Kang X. (2022). Therapeutic Effect of Exosomes Derived from Stem Cells in Spinal Cord Injury: A Systematic Review Based on Animal Studies. Front. Neurol..

[219] Feng Y., Li Y., Shen P.P., Wang B. (2022). Gene-Modified Stem Cells for Spinal Cord Injury: A Promising Better Alternative Therapy. Stem Cell Rev. Rep..

[220] Zhang B., Wang D., Li X., Yang S., Yuan H. (2021). NEP1-40-Overexpressing Neural Stem Cells Enhance Axon Regeneration by Inhibiting Nogo-A/NgR1 Signaling Pathway. Curr. Neurovasc. Res..

[221] Zhang D., Sun Y., Liu W. (2022). Motor Functional Recovery Efficacy of Scaffolds with Bone Marrow Stem Cells in Rat Spinal Cord Injury: A Bayesian Network Meta-Analysis. Spinal Cord.

[222] Haggerty A.E., Maldonado-Lasuncion I., Nitobe Y., Yamane K., Marlow M.M., You H., Zhang C., Cho B., Li X., Reddy S. (2022). The Effects of the Combination of Mesenchymal Stromal Cells and Nanofiber-Hydrogel Composite on Repair of the Contused Spinal Cord. Cells.

[223] Czyżewski W., Jachimczyk J., Hoffman Z., Szymoniuk M., Litak J., Maciejewski M., Kura K., Rola R., Torres K. (2022). Low-Cost Cranioplasty-A Systematic Review of 3D Printing in Medicine. Materials.

[224] Rezmer J., Wasilewska I., Świątek Ł. (2022). Use of 3d Printing Technology in the Treatment of Microtia and Other Outer Ear Deformities. J. Educ. Health Sport.

[225] Rezmer J., Wasilewska I., Świątek Ł. (2022). The Use of 3d Printing Technology in the Development of a Prosthetic Thumb. J. Educ. Health Sport.

[226] Zarepour A., Hooshmand S., Gökmen A., Zarrabi A., Mostafavi E. (2021). Spinal Cord Injury Management through the Combination of Stem Cells and Implantable 3D Bioprinted Platforms. Cells.

[227] Chen C., Zhao M.L., Zhang R.K., Lu G., Zhao C.Y., Fu F., Sun H.T., Zhang S., Tu Y., Li X.H. (2017). Collagen/Heparin Sulfate Scaffolds Fabricated by a 3D Bioprinter Improved Mechanical Properties and Neurological Function after Spinal Cord Injury in Rats. J. Biomed. Mater. Res. A.

[228] Sun Y., Yang C., Zhu X., Wang J.J., Liu X.Y., Yang X.P., An X.W., Liang J., Dong H.J., Jiang W. (2019). 3D Printing Collagen/Chitosan Scaffold Ameliorated Axon Regeneration and Neurological Recovery after Spinal Cord Injury. J. Biomed. Mater. Res. Part A.

[229] Li X.H., Zhu X., Liu X.Y., Xu H.H., Jiang W., Wang J.J., Chen F., Zhang S., Li R.X., Chen X.Y. (2021). The Corticospinal Tract Structure of Collagen/Silk Fibroin Scaffold Implants Using 3D Printing Promotes Functional Recovery after Complete Spinal Cord Transection in Rats. J. Mater. Sci. Mater. Med..

[230] Koffler J., Zhu W., Qu X., Platoshyn O., Dulin J.N., Brock J., Graham L., Lu P., Sakamoto J., Marsala M. (2019). Biomimetic 3D-Printed Scaffolds for Spinal Cord Injury Repair. Nat. Med..

[231] Zarepour A., Bal Öztürk A., Koyuncu Irmak D., Yaşayan G., Gökmen A., Karaöz E., Zarepour A., Zarrabi A., Mostafavi E. (2022). Combination Therapy Using Nanomaterials and Stem Cells to Treat Spinal Cord Injuries. Eur. J. Pharm. Biopharm..

[232] Somuncu D., Gartenberg A., Cho W. (2022). Investigational Therapies for Gunshot Wounds to the Spine: A Narrative Review. Clin. Spine Surg..

[233] Hachmann J.T., Yousak A., Wallner J.J., Gad P.N., Edgerton V.R., Gorgey A.S. (2021). Epidural Spinal Cord Stimulation as an Intervention for Motor Recovery after Motor Complete Spinal Cord Injury. J. Neurophysiol..

[234] Duan R., Qu M., Yuan Y., Lin M., Liu T., Huang W., Gao J., Zhang M., Yu X. (2021). Clinical Benefit of Rehabilitation Training in Spinal Cord Injury: A Systematic Review and Meta-Analysis. Spine.

[235] Hicks A.L. (2021). Locomotor Training in People with Spinal Cord Injury: Is This Exercise?. Spinal Cord.

[236] Gaojian T., Dingfei Q., Linwei L., Xiaowei W., Zheng Z., Wei L., Tong Z., Benxiang N., Yanning Q., Wei Z. (2020). Parthenolide Promotes the Repair of Spinal Cord Injury by Modulating M1/M2 Polarization via the NF-ΚB and STAT 1/3 Signaling Pathway. Cell Death Discov..

[237] Fang H., Yang M., Pan Q., Jin H.L., Li H.F., Wang R.R., Wang Q.Y., Zhang J.P. (2021). MicroRNA-22-3p Alleviates Spinal Cord Ischemia/Reperfusion Injury by Modulating M2 Macrophage Polarization via IRF5. J. Neurochem..

